# Taste and pheromonal inputs govern the regulation of time investment for mating by sexual experience in male *Drosophila melanogaster*

**DOI:** 10.1371/journal.pgen.1010753

**Published:** 2023-05-22

**Authors:** Seung Gee Lee, Dongyu Sun, Hongyu Miao, Zekun Wu, Changku Kang, Baraa Saad, Khoi-Nguyen Ha Nguyen, Adrian Guerra-Phalen, Dorothy Bui, Al-Hassan Abbas, Brian Trinh, Ashvent Malik, Mahdi Zeghal, Anne-Christine Auge, Md Ehteshamul Islam, Kyle Wong, Tiffany Stern, Elizabeth Lebedev, Thomas N. Sherratt, Woo Jae Kim

**Affiliations:** 1 Department of Cellular and Molecular Medicine, University of Ottawa, Ottawa, Canada; 2 The HIT Center for Life Sciences, Harbin Institute of Technology, Harbin, China; 3 Department of Agricultural Biotechnology, Seoul National University, Seoul, South Korea; 4 Research Institute of Agriculture and Life Sciences, Seoul National University, Seoul, South Korea; 5 Department of Biology, Carleton University, Ottawa, Canada; Universidad de Valparaiso, CHILE

## Abstract

Males have finite resources to spend on reproduction. Thus, males rely on a ‘time investment strategy’ to maximize their reproductive success. For example, male *Drosophila melanogaster* extends their mating duration when surrounded by conditions enriched with rivals. Here we report a different form of behavioral plasticity whereby male fruit flies exhibit a shortened duration of mating when they are sexually experienced; we refer to this plasticity as ‘shorter-mating-duration (SMD)’. SMD is a plastic behavior and requires sexually dimorphic taste neurons. We identified several neurons in the male foreleg and midleg that express specific sugar and pheromone receptors. Using a cost-benefit model and behavioral experiments, we further show that SMD behavior exhibits adaptive behavioral plasticity in male flies. Thus, our study delineates the molecular and cellular basis of the sensory inputs required for SMD; this represents a plastic interval timing behavior that could serve as a model system to study how multisensory inputs converge to modify interval timing behavior for improved adaptation.

## Author summary

To maximize their return on investment, male flies utilize a wide variety of sensory inputs, including memories of past sexual encounters. Therefore, when males have sufficient sexual experiences, they shorten their mating duration. The term "Shorter-Mating-Duration" (SMD) was coined to describe this behavior. SMD must be triggered by sugar and pheromone, which is detected by cells and receptors in the male forelegs. We found that these cells include male-specific sensory neurons that are tuned to collect on a male’s sexual experiences and relay that information to the brain, where it is used to determine how long to spend mating the next available mate. We hypothesize that SMD can serve as a straightforward genetic model system through which we can investigate "interval timing", the capacity of animals to distinguish between periods ranging from minutes to hours in duration.

## Introduction

From basic behaviors to complicated decisions, all animals have to make choices throughout their life to maximize their utility function [1]. The reproductive success of a male animal depends predominantly on how many of its sperm are successful in fertilizing eggs [2]. Males have a finite resource to spend on reproduction [3] and must make choices throughout their life to optimize how their resources are utilized [4]. For example, males that invest a long period of time for mating might expose themselves to the action of predators or various environmental hazards, thereby losing their competitiveness. In this regard, the ‘time investment strategy’ (the optimum allocation of time spent on given activities to achieve maximal reproductive success)’ is crucial for males. Male *Drosophila*, for instance, respond to the presence of competitors by extending the mating duration in order to guard the female and pass on their genes. Hence, female guarding has typically evolved as a tactic for males to invest their time [5].

Recent studies have revealed that male *D*. *melanogaster* shows wide variation in terms of their level of interest in females, thus providing evidence that males have also evolved to mate selectively [6]. When mating opportunities are constrained, males that show a preference for more fecund females will benefit directly by increasing the number of offspring they produce [7]. The selective mating investment exhibited by male *D*. *melanogaster* may have evolved for several reasons. First, sexual activity reduces the lifespan of males [8] due to costs arising from vigorous courtship [9], the production of ejaculates [10] and possibly also due to immunosuppression [11]. Second, repeated mating by males within a 24 h period depletes limiting components of the ejaculate [12]. Third, the quality of potential female mates is highly variable [13].

Behavioral plasticity is advantageous when specific aspects of the environment (e.g., the intensity of socio-sexual encounters) are prone to rapid and unpredictable variation [14–20]. The best-studied example of plastic behavioral responses in males is ‘longer-mating-duration (LMD)’ in which exposure to rivals before mating increases investment through mating duration [14,15,17–31].

It has been reported that previous sexual experience with females influences the mating duration of male *D*. *melanogaster* [14,19,32]; however, the neural circuits and physiology underlying this behavior have not been deeply investigated. Here, we report the sensory integration mechanisms by which sexually experienced males exhibit plastic behavior by limiting their investment in copulation time; we refer to this behavior as "shorter mating duration (SMD)."

## Results

### Sexual experiences diminish male *Drosophila*’s mating duration via chemosensory cues from females

**Fig 1:**
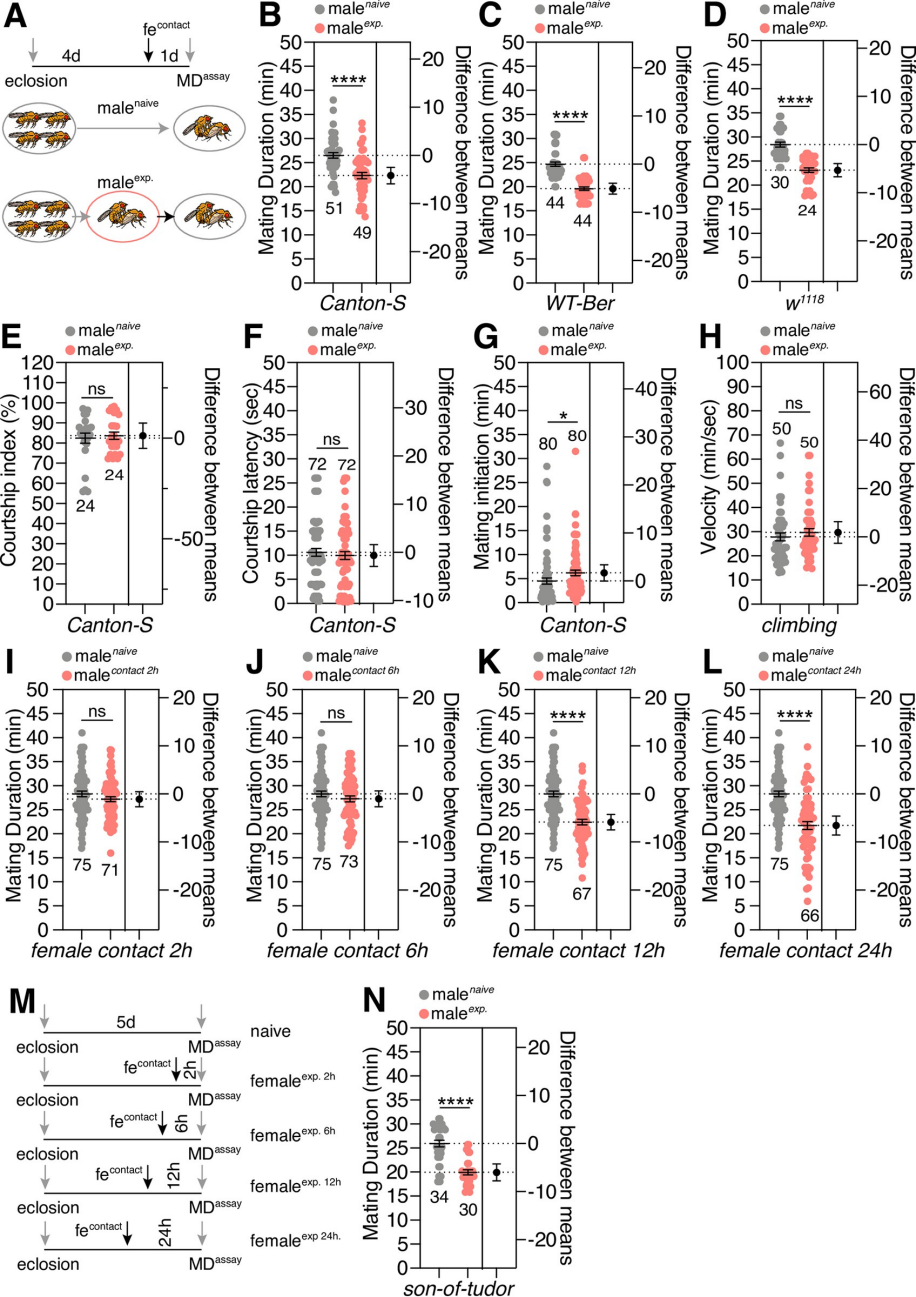
General characteristics of ‘shorter-mating-duration (SMD)’ behavior. (A) Naïve males were kept for 5 days after eclosion in groups of 4 males. Experienced males were kept for 4 days after eclosion in groups then experienced with 5 virgin females 1 day before assay; for detailed methods, see the **EXPERIMENTAL PROCEDURES**. (B) Mating duration (MD) assays of Canton-S (CS), (C) WT-Berlin, and (D) *w*^*1118*^ males. Light grey dots represent naïve males and pink dots represent experienced ones. (E) Courtship index of naïve and experienced males. See the **EXPERIMENTAL PROCEDURES** section for detailed methods. (F) Courtship latency of naïve and experienced males. See the **EXPERIMENTAL PROCEDURES** section for detailed methods. (G) Mating initiation time of naïve and experienced males. (H) The locomotion of naïve and experienced male flies was quantified as velocity by a climbing assay paradigm. (I-L) MD assays of CS males with different exposure time with females. Each group of males was reared with females for (I) 2 h, (J) 6 h, (K) 12 h or (L) 24 h. (M) A diagram showing the results of MD assays of CS males with different exposure times with females. (N) MD assays for *son-of-tudor* mutants. Genotypes are described as in a previous report [33]. Dot plots represent the MD of each male fly. The mean value and standard error are labeled within the dot plot (black lines). Asterisks represent significant differences, as revealed by the Student’s *t* test (* *p<0*.*05*, ** *p<0*.*01*, *** *p<0*.*001*). The same notations for statistical significance are used in other figures. Number signs represent significant differences, as revealed by Dunn’s Multiple Comparison Test (^#^ *p<0*.*05*). The same symbols for statistical significance are used in all other figures. See the **EXPERIMENTAL PROCEDURES** for a detailed description of the statistical analysis used in this study.

To investigate how sexual experience affects the mating duration of male *D*. *melanogaster*, we introduced virgin females to group-reared males one day before the assay (this condition is referred to as ‘experienced’ hereafter) and compared mating duration of experienced males with group-reared males that had never encountered sexual experience (this condition is referred to as ‘naïve’ hereafter) (Fig 1A). We found that the mating duration of *Canton S*, *WT-Berlin*, *Oregon-R*, and *w*^*1118*^ naïve males are significantly longer (wild type 15.7~15.8%, *w*^*1118*^ 12.4%) than that of sexually experienced males (Figs 1B–1D and S1A). Despite the fact that our previously reported LMD behavior is dependent on the *white* mutant genetic background [21], these findings show that the effect of the *white* mutant genetic background was not obvious in SMD behavior.

To test whether fatigue causes SMD behavior, we examined other behavioral repertoires of naïve and experienced male flies, such as courtship index, courtship latency, copulation latency and locomotion; there was no significant difference between experienced and naïve males (Figs 1E–1H, S1B and S1C). Thus, we conclude that potential fatigue from repetitive sexual experiences is not a causative factor for SMD behavior.

To determine the time required by males to be exposed to females in order to induce SMD behavior, we varied the exposure time of males to females and found that males significantly reduced their mating duration when their exposure to females lasted for longer than 12 h but not for less than 6 h, thus suggesting that SMD requires chronic exposure to females for longer than 6 h (Fig 1I–1M). To determine whether SMD is a reversible behavior, we separated males from females after 24 h or 48 h of sexual experience and then tested these males in a mating duration assay. We found that separating experienced males from females for 24 h was sufficient to restore the MD to the level of naïve males (S1D–S1G Fig), thus suggesting that SMD is plastic and dependent on sexual experience with females but can change over time.

To confirm the lack of effect of sperm depletion on SMD behavior, we depleted sperm prior to MD assays and found that sperm depletion did not affect SMD behavior (S1H–S1L Fig). We also tested the *son-of-tudor* males that lack germ cells and are therefore devoid of sperm [33]; we found that the *son-of-tudor* males also exhibited SMD (Fig 1N). Consistent with a previous report [34], these data suggest that sperm depletion does not cause SMD behavior in male *D*. *melanogaster*.

**Fig 2:**
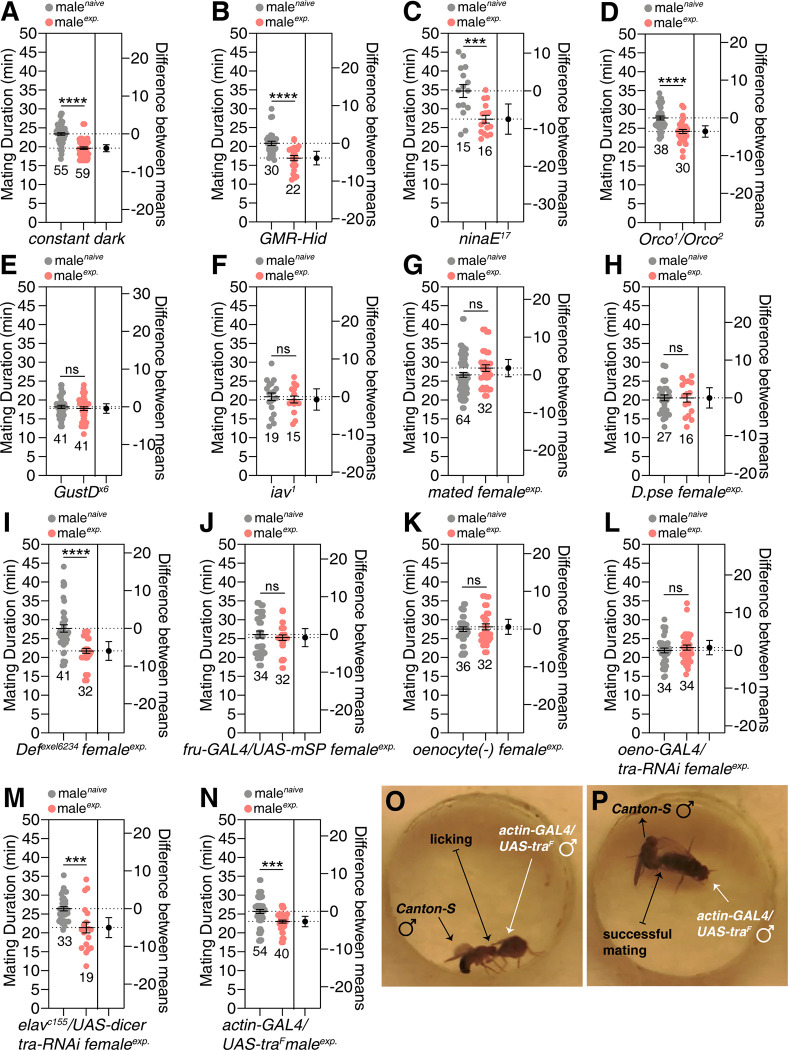
Sensory inputs required for inducing SMD behavior. (A) To test whether the vision is required for SMD, CS males were reared and sexually experienced in constant dark for 5 days (dark). (B) MD assays of *GMR-Hid* males, and blind animal. (C) MD assays of *ninaE*^*17*^ mutant males animal lacking the opsin R1-6 photoreceptors [35]. (D) MD assay of *Orco*^*1*^/*Orco*^*2*^ trans-heterozygote mutant males with defects in olfaction [87]. (E) MD assays of *GustD*^*x6*^ mutant males showing aberrant responses to sugar and NaCl [88]. (F) MD assays of *iav*^*1*^ males, the auditory and mechanosensory mutant [89]. (G) MD assays of CS males exposed to sexually experienced females 1 day before assay. To generate mated females, 4-day-old 10 CS virgin females were placed with 5-day-old 20 CS males for 6 hours and then transferred to an empty vial. These females were used for experienced females 1 day after separation. (H) MD assay of CS males experienced with *D*. *pseudoobscura* females. (I) MD assay of CS males experienced with *Df*^*exel6234*^ females, a deficiency strain that lacks the expression of the sex-peptide receptor (SPR) [90]. (J) MD assays of CS males experienced with virgin females behaving as mated females. To make virgin females behave as mated females, flies expressing *UAS-mSP* (a membrane bound form of male sex-peptide) were crossed with flies expressing *fru-GAL4* driver, as described previously [44,91]. (K) MD assays of CS males experienced with oenocyte-deleted females. To generate oenocyte-deleted females, virgin flies expressing *UAS-Hid/* crossed with flies expressing *tub-GAL80ts*, *oeno-GAL4* males; then the female progeny were kept in 22°C for 3 days. Flies were moved to 29°C for 2 days before assay to express *UAS-Hid/rpr* and kill the oenocytes in these females. The *oeno-GAL4* (*PromE(800)-GAL4*) was described previously [92]. (L) MD assays of CS males exposed to oenocyte-masculinized females. To generate oenocytes-masculinized females, flies expressing *UAS-tra-RNAi* were crossed with *oeno-GAL4* driver. (M) MD assays of CS males exposed to pan-neuronally masculinized females. To generate pan-neuronally masculinized females, flies expressing *UAS-tra-RNAi* were crossed with *elav*^*c155*^ driver [93]. (N) MD assays of CS males exposed to feminized females. To generate feminized males, flies expressing *actin-GAL4* were crossed with flies expressing *UAS-tra*^*F*^ [46,93]. (O) CS male courting with a feminized male and showing licking behavior, leading to (P) successful mating.

Next, to identify the sensory modalities that modulate SMD behavior, we tested multiple mutants with defects in various sensory modalities [21,35]. By using constant dark conditions (Fig 2A) and several mutants with impaired vision (*GMR-Hid* in Fig 2B; *ninaE*^*17*^ in Fig 2C) [21,35], impaired olfaction (*Orco*^*1*^*/Orco*^*2*^ in Fig 2D and *Orco-GAL4/UAS-KNCJ2* in S2A Fig) [36], impaired gustation (*GustD*^*x6*^ in Fig 2E and *Poxn-GAL4/Poxn-RNAi* in S2B Fig) [37,38] and impaired auditory ability and mechanosensation (*iav*^*1*^ in Fig 2F) [30,39–41], we concluded that gustatory, auditory and mechanosensory pathways are involved in generating SMD behavior but not visual or olfactory pathways. S1 Table summarizes the settings we controlled to determine the sensory modality for SMD.

Next, we attempted to identify the physiological cues from females that play important roles in the induction of SMD behavior in males. To do this, we used various genotypes of females as experienced sexual partners. Mated females and *Drosophila pseudoobscura* females did not induce SMD, thus suggesting that cues originate from virgin *D*. *melanogaster* females [42] (Fig 2G and 2H). In contrast, female *D*. *simulans*, a closely related species of *D*. *melanogaster*, can induce SMD, indicating that cues for SMD are also present in female *D*. *simulans* (S2C Fig). It is well known that *D*. *melanogaster* and *D*. *simulans* can create hybrid offspring [43]. Sexual experiences with sex peptide receptor (SPR) mutant females, who have a delayed post-mating reaction and consequently display multiple mating with males compared to wild type females [44], showed no additional influence on SMD (Fig 2I). Virgin females behave like mated females by expressing a membrane-bound version of male sex-peptide in fruitless-positive neurons, hence rejecting the male’s copulation attempt [44]. Males that were experienced with these females did not show SMD, thus suggesting that both cues from females and successful copulation are required for SMD (Fig 2J).

We produced odorless and tasteless females by killing female oenocytes *(oenocyte(-)*) [45] and females that produced a male odor *via* the masculinization of female oenocytes (*oeno-GAL4/tra-RNAi*) [46]. Males that had experience with these females did not show SMD, thus suggesting that female-specific pheromones produced by oenocytes are important cues for SMD (Fig 2K and 2L). However, males experienced with females which contained masculinized neurons showed intact SMD, thus suggesting that female forms of odor, and not female forms of neural circuits, are critical for inducing SMD behavior (Fig 2M). Interestingly, feminized males, created by overexpressing the female form of the *tra2* protein driven by a broad *GAL4* driver, can provide the cues required for SMD, thus suggesting that developmental phenotypes that are regulated by *tra2* can provide both cues from females that are sufficient to induce SMD (Fig 2N). By tracking videos of the mating assay, we were able to confirm that males exhibited a full repertoire of courtship behavior and mated successfully with oenocyte-masculinized females (S2D–S2I Fig) and feminized males (Figs 2O, 2P and S2J–S2K), thus suggesting that these experienced partners can provide a mating drive for male *D*. *melanogaster*. We also found that SMD was completely normal even when an oenocyte-masculinized female (S2L Fig) was used for assay partners, thus suggesting that SMD is independent of the genotypes of the assay partners used for mating duration assays. Collectively, these data suggest that both sexual experience and female *D*. *melanogaster*-specific odor (produced in the oenocytes) are required to induce SMD behavior. The genotypes of experienced females used to define the sensory modality for SMD are summarized in S2 Table.

**Fig 3:**
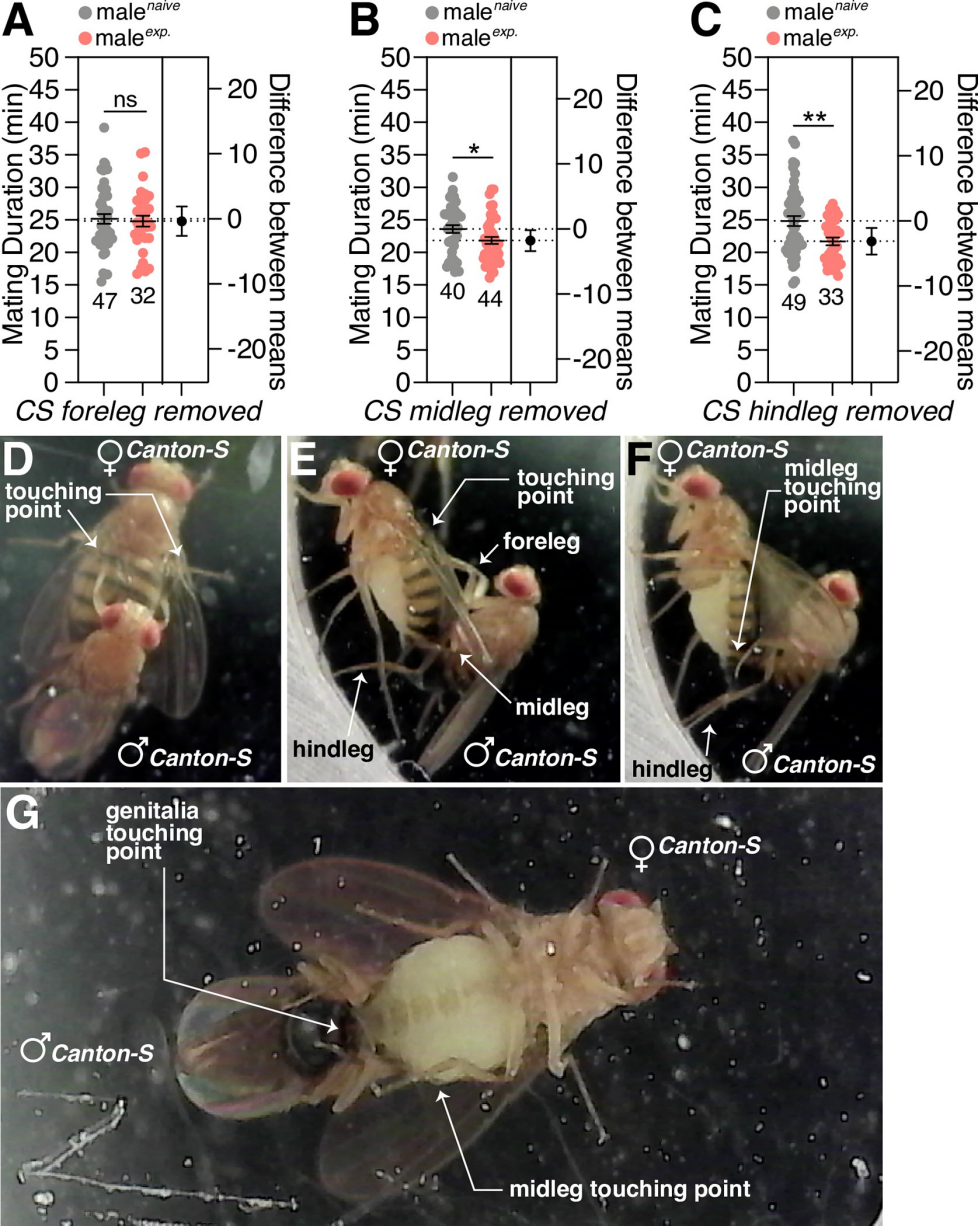
The male foreleg is crucial to detect the sensory inputs to induce SMD behavior. (A) MD assays of CS males in which the foreleg, (B) midleg, or (C) hindleg were removed 1 day before assay. Forelegs, midlegs, or hindlegs of 4-day-old males were removed by surgery and then treated as naïve or experienced for 1 d. (D) Dorsal view of the mating posture of CS males and females. The touching point of the male foreleg is marked with a white arrow. (E) Lateral view of the mating posture of CS males and females. The touching points of the male foreleg and midleg are marked with a white arrow. (F) Lateral view of the mating posture of CS males and females. The touching point of the male midleg is marked with a white arrow. (G) Ventral view of the mating posture of CS males and females. The touching points of the male midleg and genitalia are marked with a white arrow.

In flies, taste and touch signals are primarily conveyed to the brain by sensory neurons in the legs and mouthparts. To understand how sensory information for SMD is mediated *via* the legs or proboscis, we first tested the SMD behavior of males for which each pair of legs had been removed; we found that the foreleg is critical for generating SMD behavior (Fig 3A–3C). When we carefully watched the position of each pair of legs during mating, we found that the male’s foreleg touches the female body most of the time during mating; the midleg only partially touches the female body while the hind leg does not touch the female at all (Fig 3D–3G). The point at which the male’s leg touched the female body was mostly the tarsus, an area that is known to recognize taste [47] and pheromones [48] *via* chemoreception (S3A Fig). Although we cannot rule out the role of the proboscis, wings and other unidentified taste organs in the reception of stimuli for SMD behavior, our present results suggest that the male’s foreleg is the major sensor for SMD behavior.

### Gr5a-expressing sweet cells are required for SMD behavior

**Fig 4:**
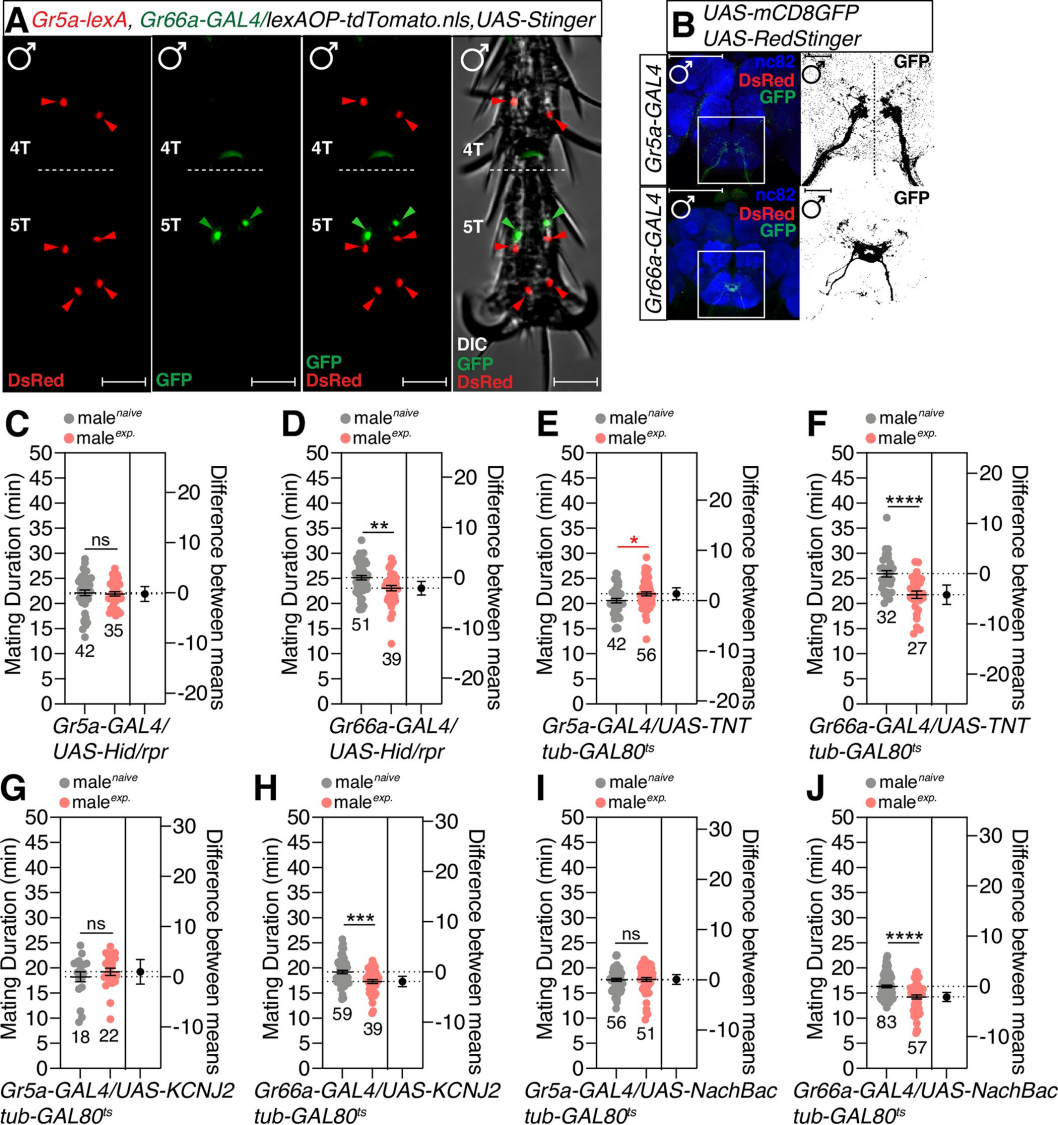
*Gr5a*-positive sugar cells are important for inducing SMD behavior in the male foreleg. (A) 4T and 5T of the male foreleg of flies expressing *Gr5a-lexA* and *Gr66a- GAL4* drivers together with *lexAOP-tdTomato* and *UAS-Stinger* were imaged live under a fluorescent microscope. Red arrows indicate *Gr5a-*positive neurons and green arrows indicate *Gr66a*-positive neurons. Scale bars represent 50 μm. (B) Brains of flies expressing *Gr5a-GAL4 or Gr66a-GAL4* together with *UAS-mCD8GFP*, *UAS-RedStinger* were immunostained with anti-GFP (green), anti-DsRed (red) and nc82 (blue) antibodies. Scale bars represent 100 μm. The right panels indicate magnified regions of the left panels that are presented as a grey scale to clearly show the axon projection patterns of gustatory neurons in the adult sub-esophageal ganglion (SOG) labeled by *GAL4* drivers. (C-D) MD assays for *GAL4* driven cell death which labelled (C) sweet cells (*Gr5a*) or (D) bitter cells (*Gr66a*) using *UAS-Hid/rpr*. (E-F) MD assays of (E) *Gr5a-* or (F) *Gr66a-GAL4* drivers for the inactivation of synaptic transmission *via* the expression of *UAS-TNT* transgene together with the *tub-GAL80*^*ts*^, such that *UAS-TNT* expression could be triggered by temperature shifts were crossed with flies expressing *tub-GAL80*^*ts*^ (G-J) electrical silencing or hyperexcitation of *Gr5a*-positive neurons abolished SMD behavior. Flies expressing (G-H) potassium channel *UAS-KCNJ2* or (I-J) bacterial voltage-gated sodium channel *UAS-NachBac* together with the *tub-GAL80*^*ts*^, such that *UAS-KCNJ2* or *UAS-NachBac* expression could be triggered by temperature shifts, were crossed with flies expressing (G and I) *Gr5a-* or (H and J) *Gr66a-GAL4* drivers. Flies were reared at 29°C for the first 2 days to strongly induce *UAS-KCNJ2* or *UAS-NachBac* expression and then transferred to 25°C for the last 3 days for the mild induction of *UAS-KCNJ2* or *UAS-NachBac* transgenes.

Of the various gustatory receptors, *Gr5a* marks cells that recognize sugars and mediate taste acceptance, whereas *Gr66*a marks cells that recognizes bitter compounds and mediates avoidance [49,50]. *Gr5a* and *Gr66a* are expressed in different cells in a sensillum of the foreleg and exhibit different sensory projections into the central brain region (Fig 4A and 4B). We found that male flies with ablated *Gr5a*-positive neurons that mediate sweet-taste detection did not exhibit SMD behavior while male flies lacking *Gr66a*-positive neurons that mediate bitter-taste detection exhibited normal SMD (Fig 4C and 4D). SMD was also impaired when we inhibited synaptic transmission *via* the expression of *TNT* in *Gr5a*-positive neurons but not in *Gr66a*-positive ones in an adult-specific manner by shifting flies bearing *tub-GAL80*^*ts*^ to restrictive temperature (29°C) after eclosion (Fig 4E and 4F). The inactivation or hyperexcitation of *Gr5a*-positive neurons, but not *Gr66a*-positive neurons, by expressing the *KCNJ2* potassium channel or *NachBac* bacterial sodium channel in an adult-specific manner using *tub-GAL80*^*ts*^, also resulted in impaired SMD (Fig 4G–4J). These data and genetic background control data (S4A–S4D Fig) suggest the cell populations of gustatory cells that mediate acceptance signals are associated with SMD behavior and that these *Gr5a*-positive neuronal populations and their neuronal activities are required for SMD.

In addition, we found that *Gr5a*-positive cells were abundantly localized in the tarsus from tarsomeres 2 (2T) to tarsomeres 5 (5T) (S4E Fig). We also found that males have more *Gr5a*-positive cells than females (S4F Fig). On average, males had 10 ± 1 neurons in the tarsus (4 cells in 5T, 2 ± 1 cells in 4T, 1 ± 1 cells in 3T, 2 cells in 2T and no cells in 1T) and 0 ± 1 cells in the tibia; however, females had 6 cells in the tarsus (4 cells in 5T and 2 cells in 4T) (S4E and S4–S4F Fig). These data suggest that *Gr5a*-positive cells show sexual dimorphism and might have a male-specific function to generate SMD.

The sexual dimorphism of sensory structure and function generates neural circuitries that are important for gender-specific behaviors. In *Drosophila*, *fruitless (fru)* is an essential neural sex determinant that is responsible for male-specific behavior [51]. To determine whether sexually dimorphic sensory neurons are involved in SMD, we used intersectional methods to genetically dissect approximately 1500 *fru* neurons into smaller subsets. We used a combination of the *fru*^*FLP*^ allele that drives FLP-mediated recombination specifically in *fru* neurons with *UAS>stop>X* genotype (X represents various reporters or effector transgenes) to express a *UAS* transgene in only those cells that were labeled by the *GAL4* driver and were also *fru*-positive; this was controlled by the FLP-mediated excision of the stop cassette (*>stop>*).

**Fig 5:**
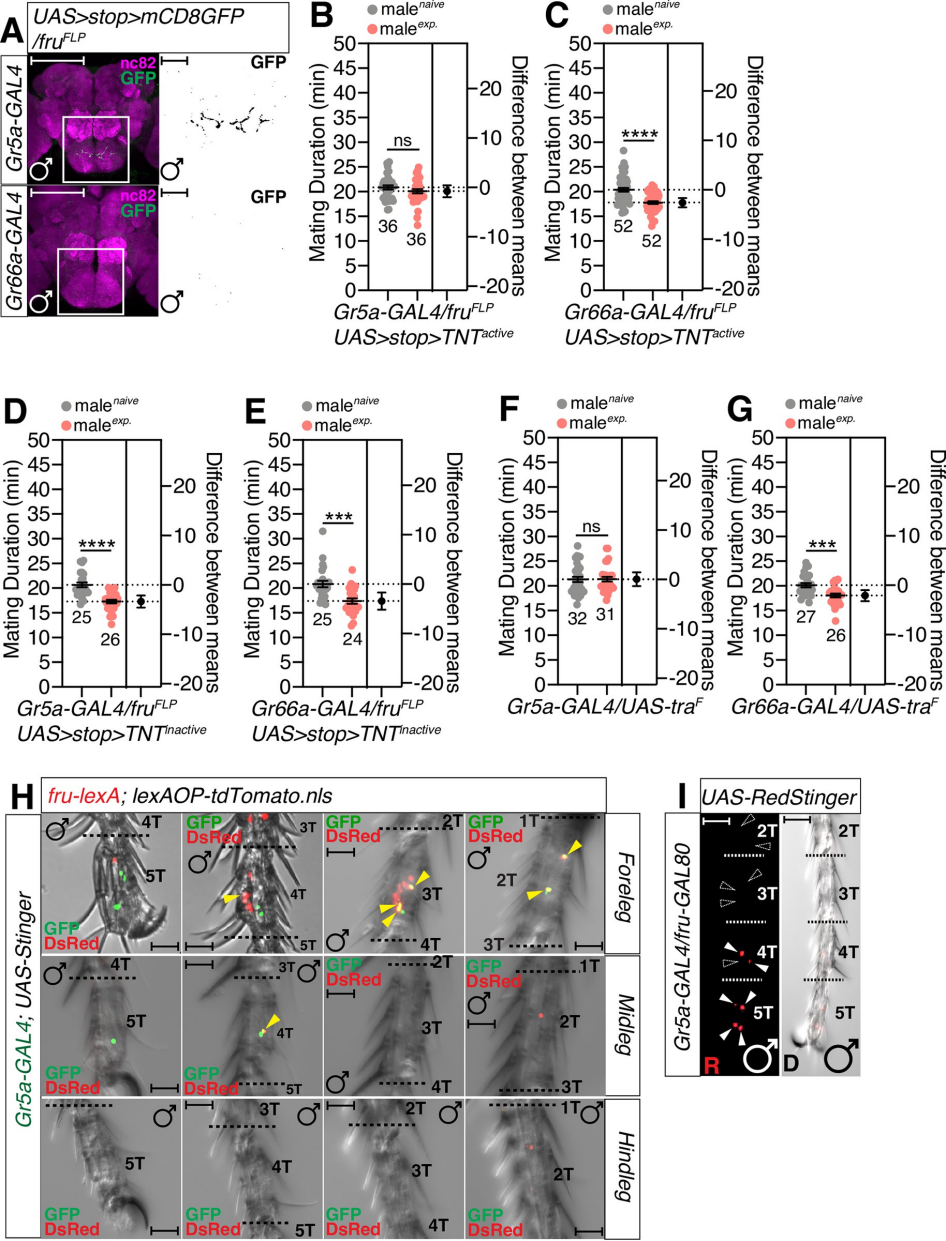
FRU-positive subsets of sugar sensing neurons in the leg are required to generate SMD. (A) Brains of male flies expressing *Gr5a-GAL4* or *Gr66a-GAL4* together with *UAS>stop>mCD8GFP; fru*^*FLP*^ were immunostained with anti-GFP (green) and nc82 (magenta) antibodies. Scale bars represent 100 μm in the colored panels and 10 μm in the grey panels. White boxes indicate the magnified regions of interest presented in the right panels. The right panels are presented as a grey scale to clearly show the axon projection patterns of gustatory neurons in the adult sub-esophageal ganglion (SOG) labeled by *GAL4* drivers. (B-C) MD assays of (B) *Gr5a-* and (C) *Gr66a-GAL4* drivers for the inactivation of synaptic transmission of *fru*-specific neurons among each *GAL4*-labelled neuron via *UAS>stop>TNT*_*active*_*; fru*^*FLP*^. (D-E) Control experiments of (B-C) with the inactive form of *UAS-TNT* using *UAS>stop>TNT*_*inactive*_*; fru*^*FLP*^. (F-G) MD assays for (F) *Gr5a-* and (G) *Gr66a-GAL4* drivers for the feminization of neurons *via UAS-tra*^*F*^. (H) Male foreleg, midleg and hindleg tarsus of flies expressing *fru-lexA* and *Gr5a- GAL4* drivers together with *lexAOP-tdTomato* and *UAS-Stinger* were imaged live under a fluorescent microscope. Red arrows indicate *Gr5a-*positive neurons and green arrows indicate *Gr66a*-positive neurons. Scale bars represent 50 μm. (I) Male foreleg of flies expressing *Gr5a-Gal4* together with *fru-GAL80*. White arrows indicate *Gr5a*-positive and *fru*-negative neurons. Dotted white arrows indicate missing neurons by adding *fru-GAL80*, as shown in **S4F Fig**.

We found that the sensory projections of a subset of *Gr5a*-positive neurons, but not *Gr66a*-positive neurons, were positive for *fruitless*, an essential neural sex-determinant that is responsible for male-specific behaviors [51] (Figs 5A and S5A). To test whether the small subset of *fru*-positive Gr5a cells is involved in SMD, we expressed tetanus toxin light chain (*UAS>stop>TNT*_*active*_*)* with *Gr5a-* or *Gr66a-GAL4* drivers along with *fru*^*FLP*^ to inhibit synaptic transmission in sexually dimorphic subsets of *fru*-positive cells. We found that SMD was abolished when *UAS-TNT* was expressed only in male-specific *Gr5a*-positive neurons (Fig 5B and 5C). As a control, we found that SMD was unaffected when we used each of these *GAL4* drivers in combination with *UAS>stop>TNT*_*inactive*_ to express an inactive form of the tetanus toxin light chain (Fig 5D and 5E). The systemic expression of a female form of *tra* cDNA (*UAS-tra*^*F*^) in a male during development is known to lead to the expression of female characteristics [52]. We found that SMD was eliminated by the feminization of *Gr5a-GAL4* labeled cells but not by the expression of *UAS-tra*^*F*^ in Gr66a-positive neuronal subsets (Fig 5F and 5G), thus suggesting that the feminization of *Gr5a*-positive neurons nullifies the male-specific sensory function of those cells to detect sensory inputs for SMD behavior. Together with genetic background control experiments (S5B–S5D Fig), these data suggest that SMD requires the male-specific role of a subset of *Gr5a*-positive neurons.

By using the genetic intersectional method [53], we found that the male foreleg contains 5–6 *Gr5a*- and *fru*-positive cells in the tarsus (1 in 4T, 2–3 in 3T and 2 in 2T) while the midleg contains 1 (1 in 4T) (Fig 5H). However, we could find one of these cells in the male proboscis (S5E Fig). We also confirmed the number and position of *Gr5a*-expressing *fru*-positive cells using *fru-GAL80* combined with *Gr5a-GAL4*, as shown in Fig 5H (Fig 5I). Together with the data arising from leg removal experiments (Fig 3), these data suggest that *Gr5a*-expressing male-specific sensory cells in the male leg provide the major sensory input for SMD generation.

### Specific sugar receptors are essential for SMD sensory information

**Fig 6:**
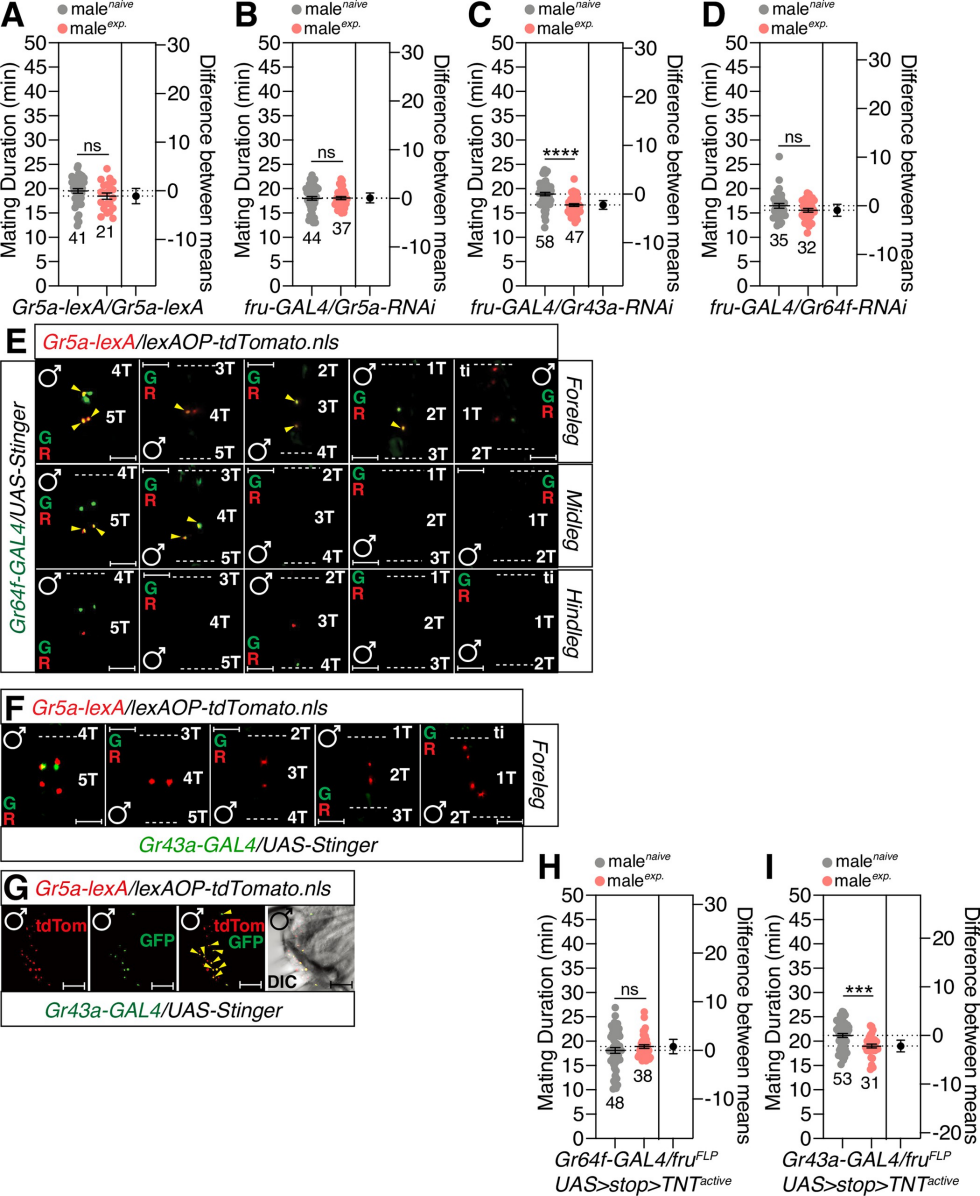
Specific sugar receptors in the male foreleg are critical for inducing SMD behavior. (A) MD assay of *Gr5a-lexA* homozygote males in which the *Gr5a* coding sequence was replaced with a sequence encoding a *lexA*::*VP16* driver [94]. (B-D) MD assays of flies expressing the *fru-GAL4* driver together with (B) *Gr5a-RNAi* (C) *Gr43a-RNAi* (D) *Gr64f-RNAi*. (E) Male foreleg (upper panels), midleg (middle panels) and hindleg (bottom panels) of flies expressing *Gr5a-lexA* and *Gr64f-GAL4* drivers together with *lexAOP-tdTomato* and *UAS-Stinger* were imaged live under a fluorescent microscope. Yellow arrows indicate *Gr5a-*positive neurons and *Gr64f*-positive neurons. Scale bars represent 50 μm. (F) Male foreleg of flies expressing *Gr5a-lexA* and *Gr43a-GAL4* drivers together with *lexAOP-tdTomato* and *UAS-Stinger* were imaged live under a fluorescent microscope. (G) Male proboscis of flies expressing *Gr5a-lexA* and *Gr43a-GAL4* drivers together with *lexAOP-tdTomato* and *UAS-Stinger* were imaged live under a fluorescent microscope. Yellow arrows indicate *Gr5a-*positive neurons and *Gr43a*-positive neurons. Scale bars represent 50 μm. (H-I) MD assays of (H) *Gr64f-* and (I) *Gr43a-GAL4* drivers for the inactivation of synaptic transmission of *fru*-specific neurons among each *GAL4*-labelled neuron *via UAS>stop>TNT*_*active*_*; fru*^*FLP*^. Tested gustatory sugar receptors were selected based on a previous study [55].

Next, we asked whether sugar receptors in the sexually dimorphic sugar sensory neurons are involved in the generation of the sensory input pathways that generate SMD. Sugars are the main group of chemicals underlying sweet taste and provide essential nutritional value for many mammals and insects [54]. Sweet taste in *D*. *melanogaster* is mediated by eight, closely related gustatory genes: *Gr5a*, *Gr61a*, and *Gr64a*-*Gr64f* [55]. The *Gr5a*^*lexA*^ allele refers to the Gr5a gene replaced by the mini-white transgene [55] results in a lack of SMD, thus suggesting that *Gr5a* itself is an important receptor for generating SMD (Fig 6A). We knocked down all known sugar receptors in *fru*-positive cells using a *fru-GAL4* driver and found that only *Gr5a* and *Gr64f* are important for the generation of SMD in male-specific *fru*-positive cells (Figs 6B–6D and S6A–S6G).

By using the genetic intersectional method, we found that *Gr5a* is co-expressed with *Gr64f* in 5T - 1T of the male foreleg and 5T - 4T in midleg (Fig 6E). However, *Gr5a* is co-expressed with *Gr64f* in 5T - 4T in the female foreleg/midleg and 5T in the female hindleg (S6H Fig). In contrast, there are no *Gr5a*-positive cells expressing the fructose sensor Gr43a [56] in the male foreleg (Fig 6F). Although no cells co-expressed Gr5a and Gr43a in the leg, several cells co-expressed Gr5a and Gr43a in the male proboscis (Fig 6G). We were unable to detect any *fru*-positive cells expressing Gr64f in the male proboscis (S6I Fig). When we expressed *UAS-TNT* only in male-specific *Gr64f*-positive neurons, we found that SMD was abolished; however, SMD remained intact in *Gr43a*-positive neurons (Fig 6H and 6I). Gr proteins are known to function as heterodimeric or multimeric complexes [57–59]. In addition, Gr64f is required broadly as a co-receptor for the detection of sugars and works together with Gr5a protein to illicit behavioral responses to trehalose [60]. Collectively, these data suggest that co-expression of the sugar receptor Gr5a and its co-receptor Gr64f in male-specific leg sensory neurons is crucial for the sensory inputs underlying SMD behavior.

### Pheromone-sensing molecules and receptors are involved in the processing of SMD sensory information

**Fig 7:**
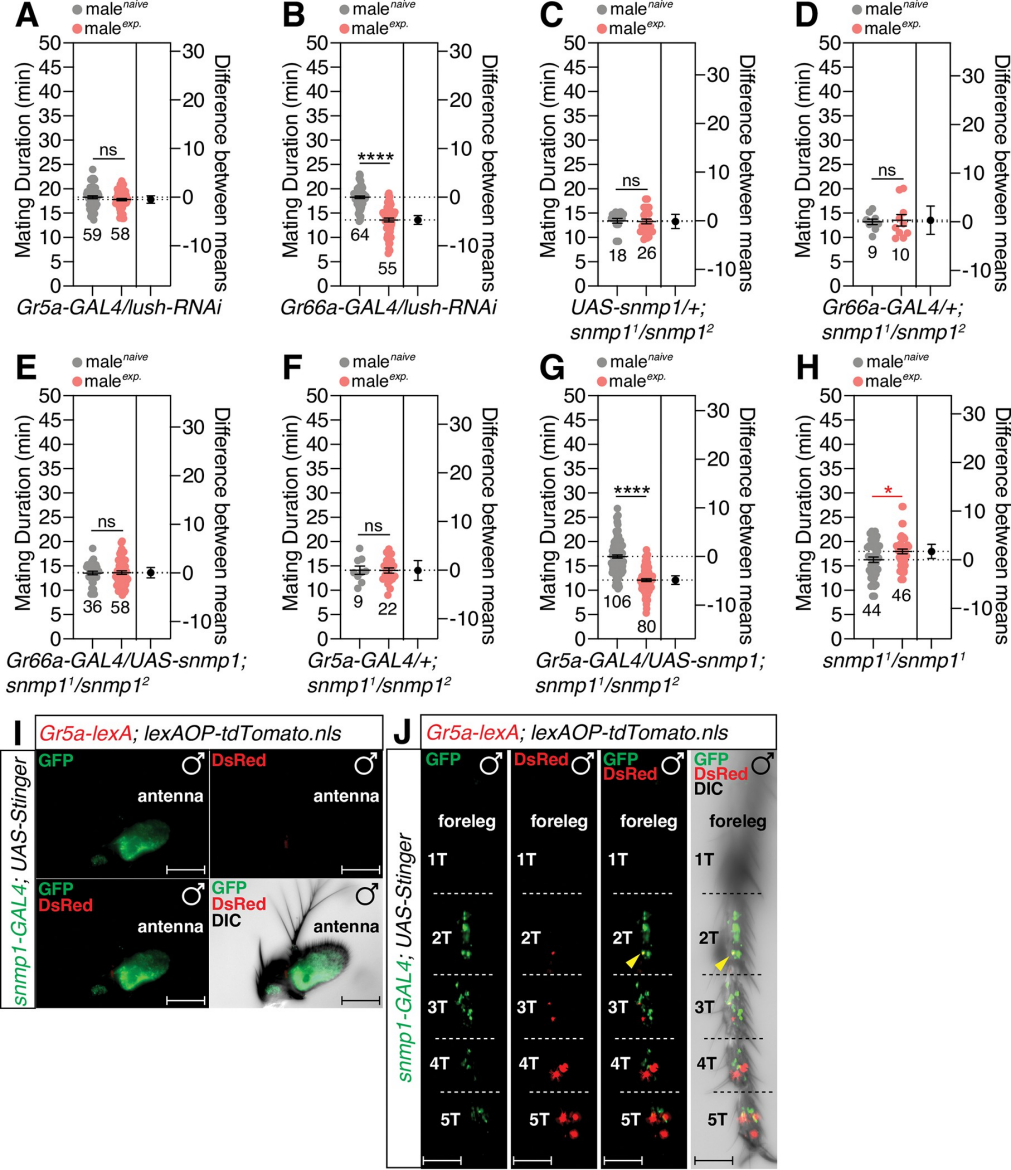
*OBPs* and *snmp1* are involved in detecting the sensory inputs for SMD behavior. (A-B) MD assays for *GAL4* mediated knockdown of LUSH *via UAS-lush-IR; UAS-dicer* (*lush-RNAi*) using (A) *Gr5a-GAL4* and (B) *Gr66a-GAL4* drivers. (C-H) MD assays of *snmp1* genetic rescue experiments. Genotypes are indicated below each graph. (I) Male antenna of flies expressing *Gr5a-lexA* and *Snmp1-GAL4* drivers together with *lexAOP-tdTomato* and *UAS-Stinger* were imaged live under a fluorescent microscope. Scale bars represent 50 μm. (J) Male foreleg of flies expressing *Gr5a-lexA* and *Snmp1-GAL4* drivers together with *lexAOP-tdTomato* and *UAS-Stinger* were imaged live under a fluorescent microscope. Yellow arrows indicate *Gr5a-*positive and *Snmp1*-positive neurons. Scale bars represent 50 μm.

Next, we tested the role of pheromone processing molecules in male legs in the generation of SMD behavior [61]. The knockdown of LUSH, an odorant-binding protein [62] in *Gr5a*-positive neurons, but not in *Gr66a*-positive neurons, led to the abolishment of SMD behavior (Fig 7A and 7B). SNMP1 is a member of the CD36-related protein family and functions as an important player for the rapid kinetics of pheromonal response in insects [63,64]. We found that the expression of Snmp1 on the *snmp1* mutant background *via* the *Gr5a-GAL4* driver, but not the *Gr66a-GAL4* driver, could rescue SMD behavior (Fig 7C–7H), thus suggesting that the expression of the pheromone sensing proteins LUSH and Snmp1 in *Gr5a*-positive gustatory neurons is critical for generating SMD behavior. By using the genetic intersectional method, we found that the male antenna contains an abundance of *snmp1*-positive cells but did not find any *Gr5a*-positive or *snmp1*-positive cells (Fig 7I). Surprisingly, we found one cell that was both *snmp1*-positive and *Gr5a*-positive in the 2T of the male tarsus (Fig 7J). SMD behavior is disrupted by *snmp1* knockdown utilizing *Gr5a-GAL4* but not *Gr66a-GAL4* (S7G–S7H Fig). Collectively, these data suggest that the expression of LUSH and SNMP1 in the male leg is crucial for sensory inputs for SMD behavior.

**Fig 8:**
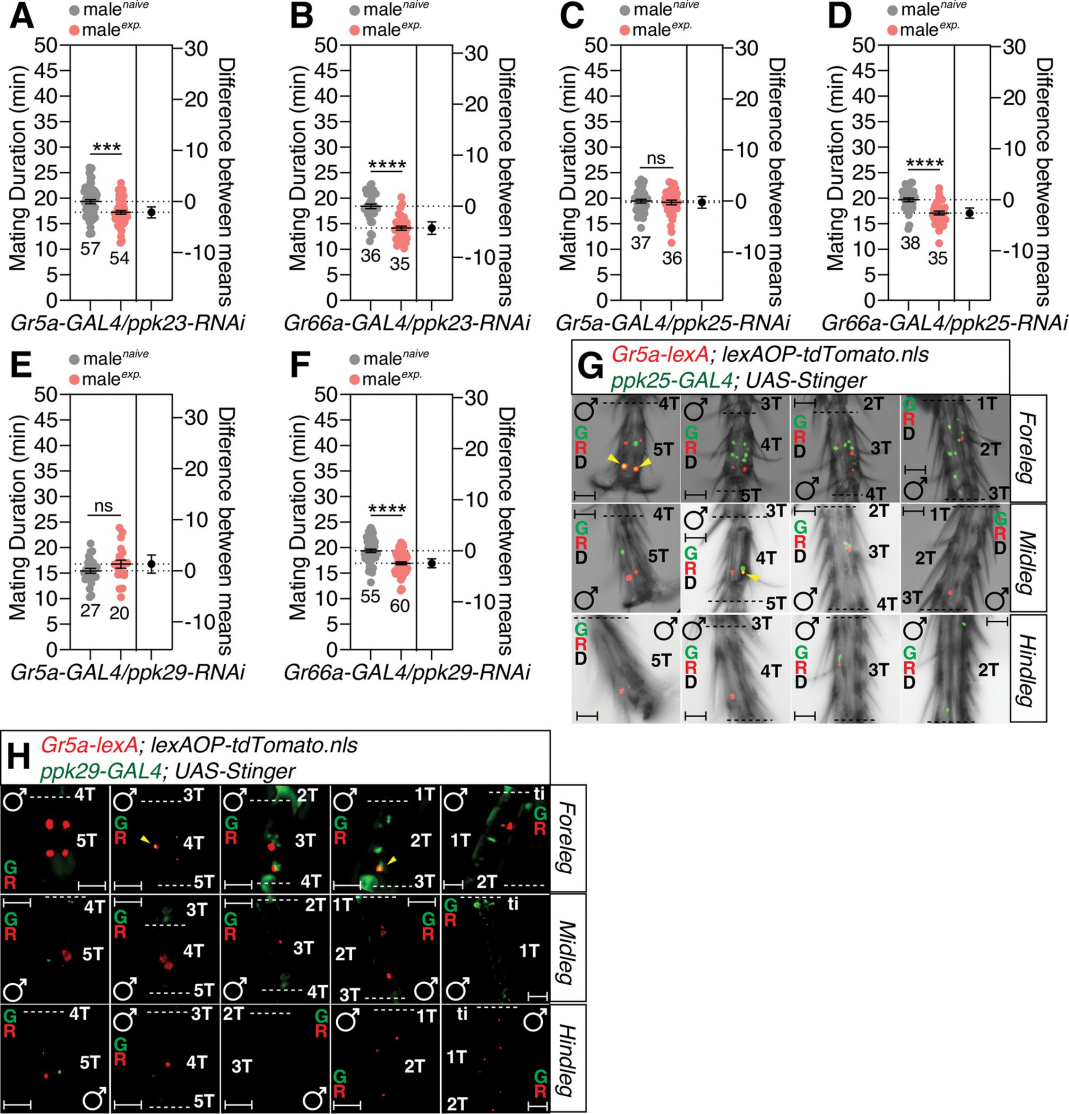
The DEG/ENaC channels *ppk25* and *ppk29* are crucial for detecting the sensory inputs for inducing SMD behavior. (A-B) MD assays for *GAL4* mediated knockdown of PPK23 *via ppk23-RNAi* using (A) *Gr5a-GAL4* and (B) *Gr66a-GAL4* drivers. (C-D) MD assays for *GAL4* mediated knockdown of PPK25 *via ppk25-RNAi* using (C) *Gr5a-GAL4* and (D) *Gr66a-GAL4* drivers. (E-F) MD assays for *GAL4* mediated knockdown of PPK29 *via ppk29-RNAi* using (E) *Gr5a-GAL4* and (F) *Gr66a-GAL4* drivers. (G) Male foreleg (upper panels), midleg (middle panels) and hindleg (bottom panels) of flies expressing *Gr5a-lexA* and *ppk25-GAL4* drivers together with *lexAOP-tdTomato* and *UAS-Stinger* were imaged live under a fluorescent microscope. Yellow arrows indicate *Gr5a-*positive and *ppk25*-positive neurons. Scale bars represent 50 μm. (H) Male foreleg (upper panels), midleg (middle panels) and hindleg (bottom panels) of flies expressing *Gr5a-lexA* and *ppk29-GAL4* drivers together with *lexAOP-tdTomato* and *UAS-Stinger* were imaged live under a fluorescent microscope. Yellow arrows indicate *Gr5a-*positive neurons and *Gr64f*-positive neurons. Scale bars represent 50 μm.

Next, we tested the importance of degenerin/epithelia Na^+^ channels (DEG/ENaC), *ppk23*, *ppk25* and *ppk29* in the excitability of pheromone-sensing cells [65,66]. By using RNAi-mediated knockdown experiments, we found that *ppk25* and *ppk29*, but not *ppk23*, are crucial for generating SMD behavior in *Gr5a*-positive cells but not *Gr66a*-positive cells (Figs 8A–8F and, S8A–S8C). By using the genetic intersectional method, we found that *ppk25* was co-expressed with *Gr5a* in 5T of the male foreleg and 4T of the midleg (Fig 8G). We also found that *ppk29* was co-expressed with *Gr5a* in 2T and 4T of the male foreleg (Fig 8H). However, we did not detect any cells that co-expressed *ppk23* and *Gr5a* in the legs of males (S8D Fig). Of the Deg/ENaC sodium channel family, *ppk28* is reported to be expressed in gustatory neurons and is known to mediate the detection of water taste [67]. By using RNAi-mediated knockdown experiments, we found that *ppk28* is dispensable for SMD behavior in *Gr5a*-positive neurons (S8H–S8J Fig). These data suggest that *ppk25*/*ppk29*, but not *ppk23*/*ppk28*, are crucial for pheromonal detection in the induction of SMD behavior in Gr5a-positive leg neurons in males.

**Fig 9:**
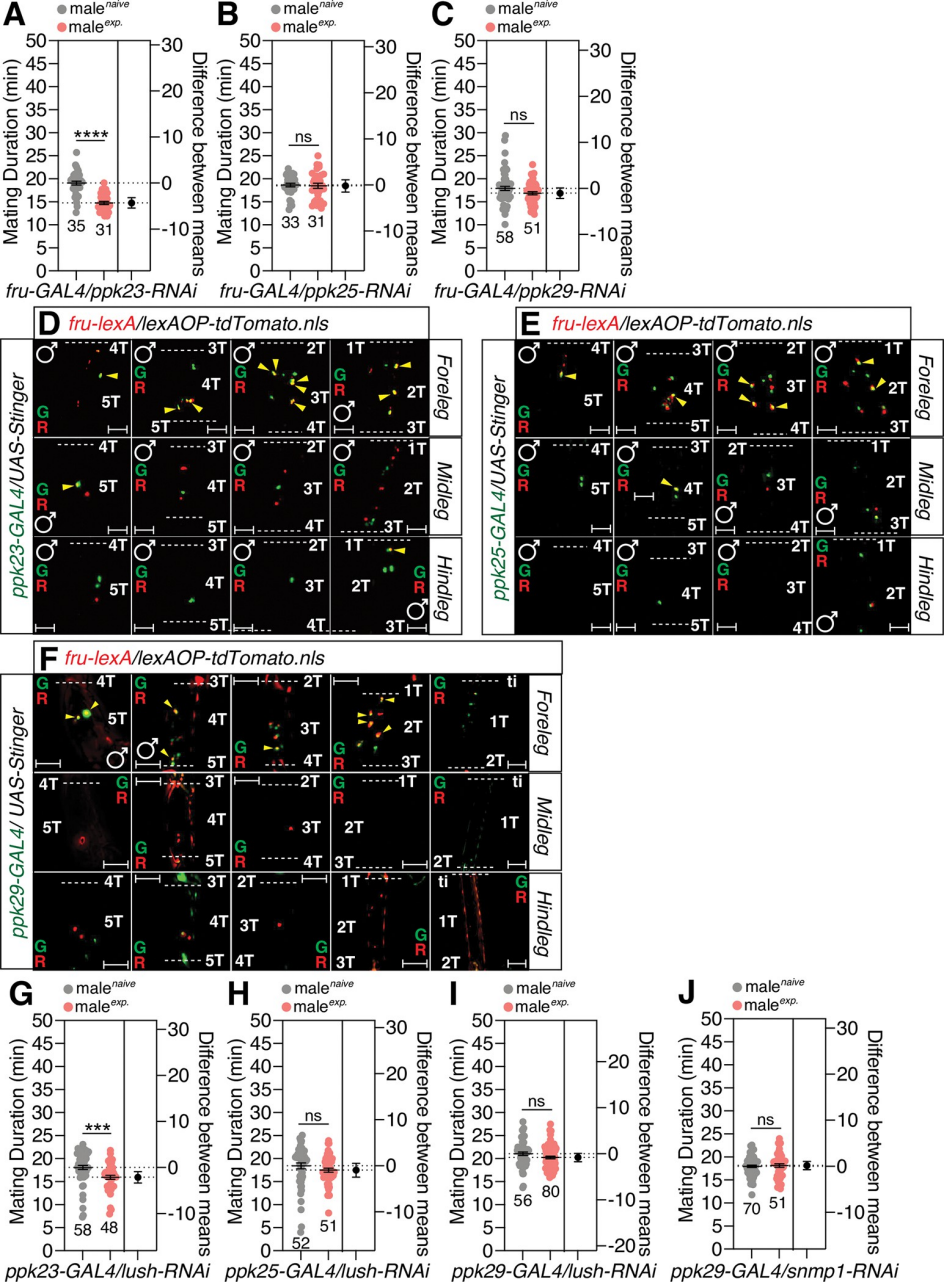
The expression of PPK25 and PPK29 in *fru*-positive sexually dimorphic cells is crucial for detecting the sensory inputs for inducing SMD behavior. (A-C) MD assays for *GAL4* mediated knockdown of (A) PPK23, (B) PPK25, and (C) PPK29 *via ppk23-RNAi*, *ppk25-RNAi*, and *ppk29-RNAi* using the *fru-GAL4* driver. (D-F) Male foreleg (upper panels), midleg (middle panels) and hindleg (bottom panels) of flies expressing *fru-lexA* and (D) *ppk23-GAL4*, (E) *ppk25-GAL4*, and (F) *ppk29-GAL4* drivers together with *lexAOP-tdTomato* and *UAS-Stinger* were imaged live under a fluorescent microscope. Yellow arrows indicate *fru-*positive and *ppk23-*, *ppk25-*, or *ppk29*-positive neurons. Scale bars represent 50 μm. (G-I) MD assays for *GAL4* mediated knockdown of LUSH *via lush-RNAi* using (G) *ppk23-GAL4*, (H) *ppk25-GAL4*, (I) *ppk29-GAL4* drivers. (J) MD assays for *GAL4* mediated knockdown of SNMP1 *via snmp1-RNAi* using the *ppk25-GAL4* driver.

Three *ppk* family members (*ppk23*, *ppk25* and *ppk29*) can sense the female pheromone 7,11-heptacosadiene [65] and express *fruitless*, a factor that is crucial for mating behavior in males [66]. By using RNAi-mediated knockdown, we found that the expression of *ppk25*/*ppk29* in *fru*-positive cells is crucial for SMD behavior, but not *ppk23* expression (Fig 9A–9C). By using the genetic intersectional method, we identified that *ppk23* was co-expressed with *fru* in 5T - 2T of the male foreleg and 2T of the hindleg (Fig 9D). We also found that *ppk25* was co-expressed with *fru* in 5T - 2T of the male foreleg and 4T of the midleg (Fig 9E) and that *ppk29* was co-expressed with *fru* in 5T - 2T of the male foreleg (Figs 9F and S9A). We also confirmed that *ppk29-GAL4* labels cells only in males and not in females (S9B and S9C Fig). These data suggest that the expression of *ppk25* and *ppk29* in *fru*-positive male-specific cells is crucial for SMD behavior.

Next, to decipher whether DEG/NaC channel-expressing pheromone sensing neurons require the function of OBP, we expressed *lush-RNAi* using *ppk23*-, *ppk25*- and *ppk29-GAL4* drivers to knockdown LUSH in each channel-expressing neuron. The knockdown of LUSH in *ppk25*- and *ppk29-GAL4* labeled cells, but not in *ppk23-GAL4* labeled cells, led to a disturbance in SMD behavior, thus suggesting that LUSH functions in *ppk25*- and *ppk29*-positive neurons to detect pheromones and elicit SMD behavior (Fig 9G–9I). The knockdown of SNMP1 in *ppk25-* or *ppk29-GAL4-* labeled neurons inhibited SMD behavior (Fig 9J and S9I Fig), thus suggesting that SNMP1 also functions in *ppk29*-positive neurons to induce SMD behavior.

The *Drosophila melanogaster* genome bears two members of the SNMP/CD36 gene family; the proteins these genes encode are expressed in distinct cells [68,69]. SNMP2 is known to contribute to gender recognition during courtship; however, its precise functional role remains unknown [69,70]. To compare the function of SNMP2 with SNMP1, a factor that is crucial for SMD behavior, we reduced the gene expression of SNMP2 in *ppk23*-, *ppk25-*, *ppk29-GAL4* expressing pheromone sensing neurons and found that SNMP2 is dispensable in these pheromone-sensing neurons for eliciting SMD behavior (S9D–S9F Fig). We also found that SNMP2 was not required for SMD behavior in *Gr5a*- and *Gr64f-GAL4* labeled sugar sensing neurons (S9G–S9H Fig). Combining with genetic control experiments (S12 and S13 Figs), all these data suggest that SNMP1, but not SNMP2, is specifically involved in pheromone detection for SMD behavior in the male leg system.

### Activation of Gr5a-positive cells is sufficient to shorten the mating duration, and this relates to calcium accumulation in these cells

**Fig 10:**
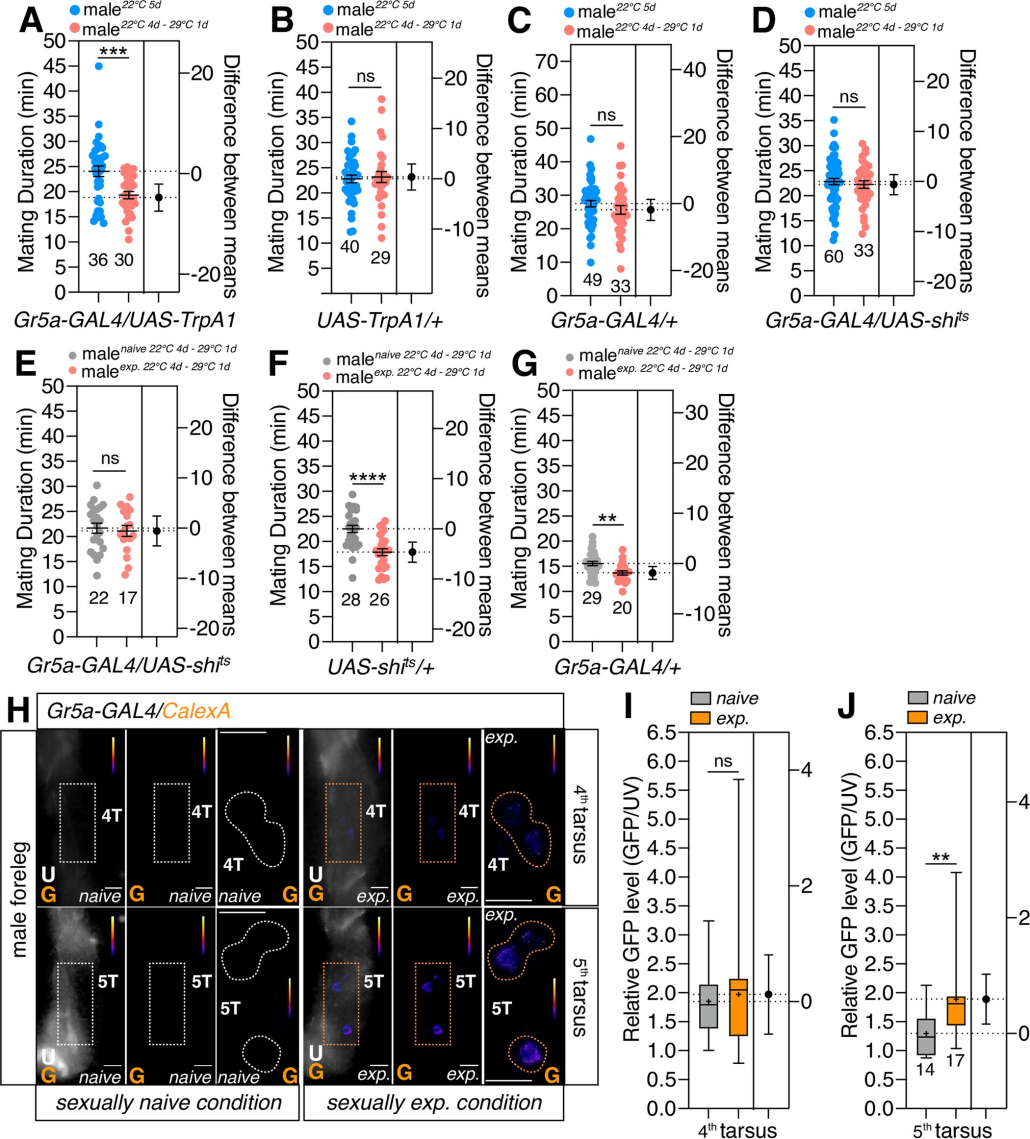
Temporal activation of Gr5a neurons induced SMD behavior without sexual experiences. (A-D) MD assay for the temporal temperature-shift of flies expressing *UAS-TrpA1* or *UAS-shi*^*ts*^ by *Gr5a-GAL4*. Genotypes are labelled below the graph. Blue groups were reared at 22°C for five days and red groups were reared at 22°C for four days and moved to 29°C overnight. (E-G) MD assay for the temporal temperature-shift of flies expressing *UAS-shi*^*ts*^ by *Gr5a-GAL4*. Genotypes are labelled below the graph. Grey groups reared at 22°C for four days and moved to 29°C for overnight. Red groups reared at 22°C for four days and moved to 29°C overnight with sexual experiences. (H) Different levels of neural activity of the 4^th^ and 5^th^ sensory neurons as revealed by the CaLexA system in naive *versus* mated male flies. Male flies expressing *Gr5a-GAL4* along with *LexAop-CD2-GFP*, *UAS-mLexA-VP16-NFAT and LexAop-CD8-GFP-2A-CD8-GFP* were dissected after at least 10 days of growth (mated male flies had 1-day of sexual experience with virgin females). GFP is pseudo-colored as “red hot”. Dashed boxes represent the magnified area of interest and show the right section of each condition. Dashed circles represent the location of *Gr5a*-positive cells. White colors represent the naïve condition while the yellow color represents the experienced condition. Scale bars represent 20 μm. (I and J) Quantification of GFP fluorescence. GFP fluorescence of the 4^th^ (I) or 5^th^ (J) tarsus was normalized to that in auto-fluorescence. The conditions of flies are described above: naïve, naïve male flies; exp., male flies with sexual experience. Bars represent the mean of the normalized GFP fluorescence level with error bars representing the SEM. Asterisks represent significant differences, as revealed by the Student’s *t* test and ns represents non-significant difference (*p < 0.05, **p < 0.01, ***p < 0.001).

To determine whether the temporal activation of *Gr5a*-positive neurons may generate SMD behavior in the absence of sexual experiences, we expressed the heat-sensitive *Drosophila* cation channel *TrpA1* in *Gr5a*-positive cells and then transferred the experimental group only to the activation temperature (29°C). Surprisingly, the flies expressing *TrpA1* in *Gr5a*-positive neurons at the activation temperature showed a shorter mating duration than those that remained at 22°C (Fig 10A). Neither the genetic control (Fig 10B and 10C) nor the flies expressing *shi*^*ts*^ that could disrupt synaptic transmission in a temperature-sensitive fashion (Fig 10D) showed changes in their mating duration between 22°C and 29°C. These findings indicate that the stimulation of *Gr5a*-positive neurons is sufficient to generate SMD behavior.

By using the expression of *shi*^*ts*^ with *Gr5a-GAL4*, we then attempted to inhibit the synaptic transmission of *Gr5a*-positive neurons during sexual experiences. We discovered that inhibiting *Gr5a*-positive neurons during sexual interactions by increasing the temperature to 29°C could impair SMD behavior (Fig 10E). The genetic control exhibited no such result (Fig 10F and 10G). These findings imply that the neural stimulation of *Gr5a*-positive neurons during the sexual experiences is a crucial trigger for SMD behavior.

To determine whether neuronal activities undergo alterations in neurons associated with SMD, we utilized the CaLexA (calcium-dependent nuclear import of *LexA*) system [71]. This system is based on the activity-dependent nuclear import 1of the transcription factor nuclear factor of activated T cells (*NFAT*). Because SMD needs at least 6–12 h of sexual interaction, repeated sensory inputs might theoretically lead to the buildup of the modified transcription factor within the nucleus of activated neurons *in vivo*. Indeed, sexual encounters affected the neural activity of some *Gr5a-GAL4*-labeled neurons. Male flies with sexual experience and carrying *Gr5a-GAL4* and *LexAop-CD2-GFP; UAS-mLexA-VP16-NFAT*, *LexAop-CD8- GFP-2A-CD8-GFP* exhibited strong fluorescence in the 5^th^ tarsus following an overnight sexual experience. In contrast, no similar signals were identified in males with no prior experience. In contrast to *Gr5a*-positive neurons in the 5th tarsus, cells in the 4^th^ tarsus did not exhibit a significant increase in GFP fluorescence (Fig 10H–10J), thus indicating that sexual encounters change the neuronal activity of *Gr5a* cells in the 5^th^ tarsus.

### SMD is an evolutionary adaptive trait

To explore the adaptive value of SMD, we developed a theoretical model to test the adaptive value of SMD behavior based on the marginal value theorem [72,73] (S1 Box). This model assumes that (i) the differences in mating duration occur largely due to the variation in post-ejaculation period (mate guarding) [15,19] and (ii) both the benefits and costs of mate guarding accumulate over time, but with different aspects.

**Fig 11:**
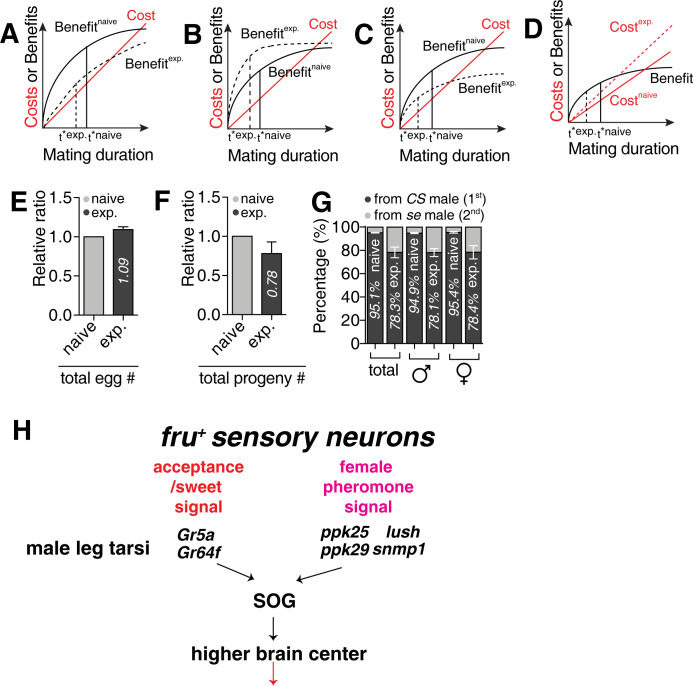
Adaptive benefits of SMD behavior. (A)-(D) show the four different scenarios by which SMD can evolve (see **S1 Box**). SMD can evolve when α gets larger (A), β gets smaller (B), γ gets larger (assuming β/α < γ < e*β/α) (C) and/or γ gets smaller (assuming γ > e*β/α) (D). (Ε) Relative ratio of total egg number comparing the eggs produced by females mated with naïve males to the eggs produced by females that mated with experienced males. (naïve = control bar for comparing, exp. = eggs from naïve males/eggs from exp. males). (F) Relative ratio of total progeny number comparing the progeny produced by the females mated with naïve males to the progeny produced by females mated with experienced males. (naïve = control bar for comparing, exp. = progeny from naïve males/progeny from exp. males). (G) Percentage of progeny originated from *sepia* (*se*) male *versus CS* male. *se* male was introduced to *se* female as first mate then followed by *CS* males as the second mate. The eye color of progeny was counted and interpreted as the source of the father; for detailed methods, see the **EXPERIMENTAL PROCEDURES**. (H) Summary of this study showing the multisensory inputs modulating SMD behavior.

The benefit refers to the number of eggs fertilized by the guarding male while the costs refer to the guarding-associated potential costs such as increased predation risk or the loss of opportunities for other forms of mating or foraging activity [74]. The model suggests that shortened mating durations can be preferred in experienced males if (1) experienced males can fertilize a fewer number of eggs in total than naïve males (Fig 11A) and that the rate of fertilization is (2) faster (Fig 11B) or (3) slower (Fig 11C) for experienced males while the total number of eggs that can be fertilized remains the same as for naïve males, and/or 4) the costs accumulate faster in experienced males (Fig 11D). Next, we empirically tested which scenario(s) could explain the observed SMD behavior. We focused on testing scenarios 1–3 but not 4, firstly because it was hard to identify a rationale for how the costs of mate guarding differ between experienced and naïve males and secondly, to experimentally manipulate the costs.

We found that the total number of eggs produced by females that mated with experienced males was comparable to those that mated with naïve males (Fig 11E); however, the number of progeny from the experienced males was significantly lower than those from naïve males (Fig 11F). When females that mated with an experienced or naïve male were subsequently introduced to another male after 24 hours, the number of progenies arising from the experienced males was also significantly fewer than those from the naïve males (Figs 11G and S11A). This suggests that (i) the number of sperm or seminal proteins from experienced males for fertilization in a given period of time was lower than that from naïve males [32,75] or ii) females reduced the use of sperm from experienced males for fertilization when they had a choice. These results support scenario 1 and potentially scenario 3 in that SMD has evolved because the reproductive payoffs of experienced males through mate-guarding are consistently lower than those of naïve males.

## Discussion

Our study provides new lines of evidence that male flies invest less time for mating duration when they are sexually experienced. Males retain a memory of sexual experience for several hours and economize mating duration accordingly (Fig 1). This behavior relies primarily on gustatory input from the male forelegs, indicating that contact chemoreception is required for SMD induction (Figs 2 and 3). Sugar cells expressing *Gr5a*, but not bitter cells expressing *Gr66a*, were found to be involved in the induction of SMD (Fig 4). We also found that male-specific, *fru-*expressing *Gr5a*-positive sensory neurons are required to recognize the presence of females (Fig 5). Sugar receptors such as *Gr5a/Gr64f*, but not fructose sensor *Gr43a*, are important for the sensory inputs required for SMD behavior (Fig 6). Chemosensory proteins such as *lush* and *SNMP1*, as well as female pheromone receptors (DEG/ENaC channel *ppk25* and *ppk29*) are important for generating SMD (Figs 7, 8 and 9). We discovered that temporal stimulation of Gr5a neurons reduces mating duration, which is related to calcium accumulation (Fig 10). Using both theoretical and empirical approaches, we further showed that SMD represents the adaptive behavioral plasticity of male flies (Fig 11).

Previous research by our group and others demonstrated that past exposure to rivals lengthens mating duration, a characteristic known as longer-mating-duration (LMD) [14–23,76]. The two behavioral circuits for LMD and SMD might have evolved independently since they use different sensory cues for detecting ‘rivals’ or ‘females’ for ‘sexual competition’ or ‘mating investment’, respectively. We propose that multisensory inputs from male forelegs detect the chemical signals from the female body and contribute to the determination of mating investment in male *Drosophila melanogaster* (S10A Fig and Fig 11H). The visual inputs from the male’s compound eye are the most crucial sensory cue to generate LMD [22]; however, multisensory inputs from the foreleg are required to induce SMD (Fig 11H). To confirm that LMD does not require female pheromone signaling, we reduced the expression of the female pheromone receptor *ppk29* in all neuronal populations using RNAi-mediated knockdown experiments and found that the neuronal expression of *ppk29* is only essential for SMD but not for LMD behavior (S11B–S11C Fig). Consistent with our previous report on the different neural circuitry for LMD and SMD [23], these data clearly show that male flies use different sensory modalities to generate LMD or SMD, respectively.

In our sugar receptor screening for SMD behavior, we found that only *Gr5a* and *Gr64f* were required for SMD behavior (Fig 6A, 6B, and 6D). The other known sugar receptors (*Gr61a*, *Gr64a*, *Gr64b*, *Gr64c*, *Gr64d*, and *Gr64e*) are not required for SMD behavior (S6A–S6G Fig). Fujii et al reported the expression code for specific sweet neurons in labial palp and tarsal sensilla [55]. In this code, *Gr5a*- and *Gr64f*-positive but *Gr43a*-negative neurons are referred to as “f4b”, “f4s”, “f5s”, and “f5b”. In the foreleg, the hair cells expressing *Gr43a* do not express *Gr5a* [55]. Gr43a is the fructose sensor and is co-expressed with Gr61a [56]. In summary, we suggest that the sugar receptors *Gr5a* and *Gr64f* in *fruitless*-positive cells provide crucial sensory information for SMD behavior.

It is known that the type of neurons expressing *ppk23*, *ppk25*, and *ppk29* is referred to as a “female” cell (F cell) from its responses to female aphrodisiac pheromones; the other type of neurons, expressing *ppk23 and ppk29* but not *ppk25*, is referred to as the “male” cell (“M” cell) from its response to male anti-aphrodisiac pheromones. M and F cells both express *fruitless* gene [66]. SMD requires female pheromonal inputs through the contact chemoreception pathway in males (Fig 2K–2M). We also identified that there are *ppk25*- and/or *ppk29*-positive neurons among *Gr5a*-positive sugar detecting neurons (Fig 8A–8H). Thus, we hypothesize that F cells, which can detect sugar taste, are responsible for SMD behavior. Several groups have reported that *ppk23*-expressing cells respond to pheromones but not to water, salt, or sugars; in addition, this response is abolished by the mutation of either *ppk23* or *ppk29* [48,77,78]. Genetic rescue studies revealed that although all three subunits are co-expressed and function in the gustatory cells required for the activation of courtship by pheromones, each has a non-redundant function within these cells [66]. Thus, we suggest that the *ppk25* and *ppk29* receptors expressed in *fruitless*-positive “F cells” are critical for detecting female body pheromones *via* contact chemoreception and generating SMD behavior.

**Fig 12:**
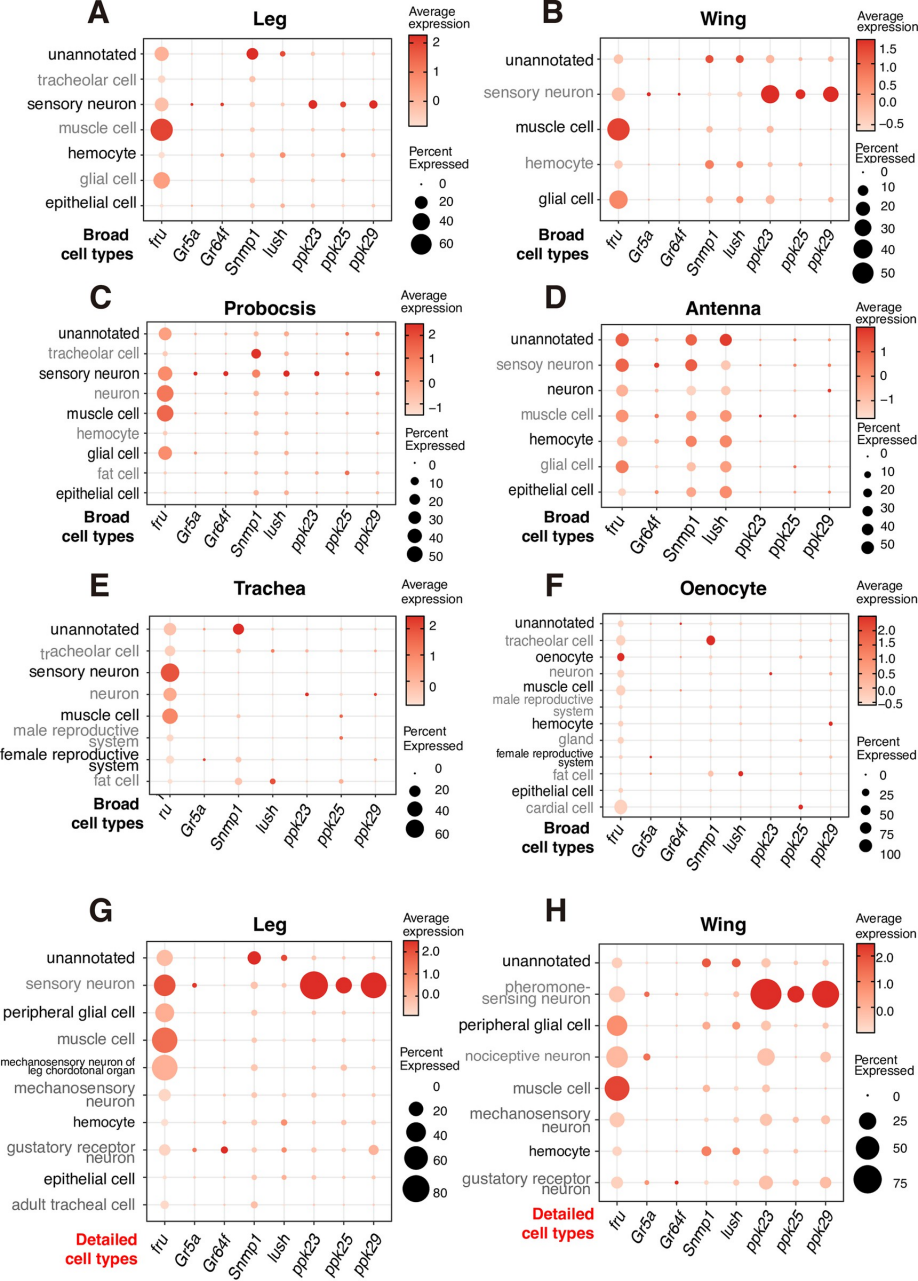
Dot plot of the 8 genes involved in SMD in each cell type in different tissues (A-H) The size of dots represents the percentagew of cells in one cell type expressing the gene of interest; the intensity of color reflects the average scaled expression. We used broad annotation for (A-F) and detailed one for (G) and (H).

One of the findings of this report is that *Gr5a*-positive taste neurons also express the female pheromone receptors *ppk25* and *ppk29*. To further validate our experimental data, we made use of a scRNA sequencing dataset of fruit flies that is available on the SCope website [79]. We reviewed the expression levels of essential marker genes for SMD behavior in several sensory organs, including the leg, wing, proboscis, antenna, trachea, and oenocyte, and concluded that these genes are expressed comparably in the leg and wing, but not in other sensory organs (Fig 12A–12F). In addition, we discovered that *Gr5a* and *Gr64f* are expressed in gustatory receptor neurons and other sensory neurons in the leg, which are pheromone-sensing neurons in the wing, as we continued to divide cell types (Fig 12G and 12H). Comparable to the leg, the wing may be an organ that can receive signals from females. Recent research found that pheromone sensing *ppk29* and *ppk23* were significantly expressed in the wing [80], thus indicating that the wing is also an intriguing organ for pheromone sensing function and may contribute to the mating behavior of males. Future research will investigate the potential role of the wings in SMD behavior.

In summary, we report a novel sensory pathway that controls mating investment related to sexual experiences in *Drosophila*. Since both LMD and SMD behaviors are involved in controlling male investment by varying the interval of mating, these two behavioral paradigms will provide a new avenue to study how the brain computes the ‘interval timing’ that allows an animal to subjectively experience the passage of physical time [81–86].

## Materials and methods

### Fly rearing and strains

*Drosophila melanogaster* were raised on cornmeal-yeast medium at similar densities to yield adults with similar body sizes. Flies were kept in 12 h light: 12 h dark cycles (LD) at 25°C (ZT 0 is the beginning of the light phase, ZT12 beginning of the dark phase) except for some experimental manipulation (experiments with the flies carrying tub-GAL80ts). Wild-type flies were Canton-S. To reduce the variation from genetic background, all flies were backcrossed for at least 3 generations to CS strain. All mutants and transgenic lines used here have been described previously.

We are very grateful to the colleagues who provided us with many of the lines used in this study. We obtained the following lines from Dr. Joel D. Levine and Joshua J. Krupp (University of Toronto, Canada): *PromE(800)-GAL4* (*oeno-GAL4* in this study); from Dr. Barry Dickson (HHMI Janelia Research Campus, USA): *UAS[stop]mCD8GFP; fruFLP*, *UAS[stop]nsybGFP; fruFLP*, *UAS[stop]TNTactive; fruFLP*, *fru-GAL4*; from Dr. Toshiro Aigaki (Tokyo Metropoitan University, Japan): *UAS-mSP*; from Dr. Martin Heisenberg (Universität Würzburg, Germany): *WT Berlin*, *ninaE17*; from Dr. Michael Gordon (University of British Columbia, Canada): *Gr5a-lexA*, *Gr64f-lexA*, *ppk23-GAL4*, *ppk25-GAL4*, *ppk29-GAL4*.

The following lines were obtained from Bloomington Stock Center (#stock number): *Orco*^*1*^ (#23129), *Orco*^*2*^ (#23130), *UAS-tubGAL80*^*ts*^ (#7018), *Df(1)*^*Exel6234*^ (#7708), *UAS-tra*^*F*^ (#4590), *GustD*^*x6*^ (#8607), *Gr66a-GAL4* (#28801), *UAS-mCD8GFP* (#5130), *UAS-RedStinger* (#8547), *snmp1*^*1*^ (#25043), *snmp1*^*2*^ (#25042), *UAS-snmp1* (#25044), *Snmp2-RNAi* (#51432), *UAS-TNT* (#28997), *UAS-dicer* (#24650, #24651), *tra-RNAi* (#28512), *lush-RNAi* (#31657), *tud*^*1*^ (*son-of-tudor* males were the sons of Oregon R males and virgin tudor females: tud1 bw sp/tud1 bw sp) (#1786), *lexAop -tdTomato*.*nls*, *UAS-Stinger* (#66680), *Gr5a-RNAi* (#31282), *Gr43a-RNAi* (#64881), *Gr61a-RNAi* (#54030), *Gr64c-RNAi* (#36734), *ppk23-RNAi* (#28350), *ppk25-RNAi* (#27088), *ppk29-RNAi* (#27241), *fru-lexA* (#66698), *Gr64f-GAL4* (#57668), *se*^*1*^ (*sepia* mutants for fecundity test, #1668), *elav*^*c155*^*; UAS-Dcr-2* (#25750), CalexA (#66542), *UAS-TrpA1* (#26264), *UAS-shi*^*ts*^ (#44222), *Poxn-GAL4* (#66685), *Poxn-RNAi* (#26238); from Vienna Drosophila Stock Center: *Gr5a-RNAi* (#v13730), *Gr43a-RNAi* (#v39518), *Gr61a-RNAi* (#v106007), *Gr64a-RNAi* (#v103342), *Gr64b-RNAi* (#v42517), *Gr64d-RNAi* (#v29422), *Gr64e-RNAi* (#v109176), *Gr64f-RNAi* (#v105084). Following transgenic stocks are available from Korea Drosophila Resource Center (KDRC): *UAS[stop]TNTinactive; fruFLP* (1124).

### Mating duration assays

Mating duration assay was performed as previously described [21,22]. For naïve males, 4 males from the same strain were placed into a vial with food for 5 days. For experienced males, 4 males from the same strain were placed into a vial with food for 4 days then eight CS virgin females were introduced into vials for last 1 day before assay. Five CS females were collected from bottles and placed into a vial for 5 days. These females provide both sexually experienced partners and mating partners for mating duration assays. At the fifth day after eclosion, males of the appropriate strain and CS virgin females were mildly anaesthetized by CO2. After placing a single female in to the mating chamber, we inserted a transparent film then placed a single male to the other side of the film in each chamber. After allowing for 1 h of recovery in the mating chamber in a 25°C incubator, we removed the transparent film and recorded the mating activities. Only those males that succeeded to mate within 1 h were included for analyses. Initiation and completion of copulation were recorded with an accuracy of 10 sec, and total mating duration was calculated for each couple. All assays were performed from noon to 4 pm. We conducted blinded studies for every test.

### Sperm depletion from males

To deplete sperm from males, 40 virgin *Def*^*exel6234*^ females which lacks SPR and shows multiple mating with males [44] were placed in a vial containing four CS males for indicated time (2 h, 4 h, 8 h, and 24 h).

### Courtship assays

Courtship assay was performed as previously described [95], under normal light conditions in circular courtship arenas 11 mm in diameter, from noon to 4 pm. Courtship latency is the time between female introduction and the first obvious male courtship behavior such as orientation coupled with wing extensions. Once courtship began, courtship index was calculated as the fraction of time a male spent in any courtship-related activity during a 10 min period or until mating occurred. Mating initiation is the time after male flies successfully mounted on female.

### Locomotion assays

For climbing assay, individual flies were placed in a 15 ml falcon tube (Fisher Scientific) and were gently tapped to the bottom of the tube. The time taken for the flies to climb 8 cm of the tube wall was recorded. Each fly was tested 5 times. Other than a single instance, all flies were seen to reach the target height within 2 min, which was the experimental cut-off time. Velocity was obtained by dividing the lines (mm) a fly crossed (distance walked) by time (sec) a fly reached the line of the tube. For horizontal (spontaneous) locomotor activities, a single fly was first brought to the middle of the column by gentle shaking and then the fly movement was constantly monitored for 5 min and recorded. Total fraction of time flies walked during 5 min was calculated and number of stops during 5 min was also counted then calculated [96].

### Immunostaining and antibodies

As described before [22], brains dissected from adults 5 days after eclosion were fixed in 4% formaldehyde for 30 min at room temperature, washed with 1% PBT three times (30 min each) and blocked in 5% normal donkey serum for 30 min. The brains were then incubated with primary antibodies in 1% PBT at 4oC overnight followed with fluorophore-conjugated secondary antibodies for 1 hour at room temperature. Brains were mounted with anti-fade mounting solution (Invitrogen, catalog #S2828) on slides for imaging. Primary antibodies: chicken anti-GFP (Aves Labs, 1:1000), rabbit anti-DsRed express (Clontech, 1:250), mouse anti-Bruchpilot (nc82) (DSHB, 1:50), mouse anti-PDF (DSHB, 1:100). Fluorophore-conjugated secondary antibodies: Alexa Fluor 488-conjugated goat anti-chicken (Invitrogen, 1:100), Alexa Fluor 488-conjugated donkey anti-rabbit (Invitrogen, 1:100), RRX-conjugated donkey anti-rabbit (Jackson Lab, 1:100), RRX-conjugated donkey anti-mouse (Jackson Lab, 1:100), Dylight 649-conjugated donkey anti-mouse (Jackson Lab, 1:100).

### Quantitative analysis of GFP fluorescence

To quantify the calcium level in leg sensory neurons, we measured fluorescence intensity using the measure tool of ImageJ (National Institutes of Health, http://rsb.info.nih.gov/ij).). Fluorescence was quantified in a manually set region of interest (ROI) of the 4^th^ or 5^th^ tarsus. To compensate for differences in fluorescence between different ROI, GFP fluorescence for CaLexA was normalized to autofluorescence, and then the fluorescence of ROI was quantified using the measure tool of ImageJ. All specimens were imaged under identical conditions.

### Statistical analysis

Statistical analysis of mating duration assay was described previously [21,22]. More than 36 males (naïve or experienced) were used for mating duration assay. Our experience suggests that the relative mating duration differences between naïve and experienced condition are always consistent; however, both absolute values and the magnitude of the difference in each strain can vary. So, we always include internal controls for each treatment as suggested by previous studies [30]. Therefore, statistical comparisons were made between groups that were naively reared or sexually experienced within each experiment. As mating duration of males showed normal distribution (Kolmogorov-Smirnov tests, p > 0.05), we used two-sided Student’s t tests. We summarized the normality and lognormality test of mating duration in S1M–S1N Fig and **S3 Table**. Each figure shows the mean ± standard error (s.e.m) (*** = p < 0.001, ** = p < 0.01, * = p < 0.05). All analysis was done in GraphPad (Prism). Individual tests and significance are detailed in figure legends.

When we compare the difference of mating duration in experiments without internal control built in, we always performed control experiments of wild type for each independent experiment for internal comparison. And in this case, we analyzed data using ANOVA for statistically significant differences (at a 95.0% confidence interval) between the means of mating duration for all conditions. If a significant difference between the means was found by Kruskal-Wallis test, then the Dunn’s Multiple Comparison Test was used to compare the mean mating duration of each condition to determine which conditions were significantly different from condition of interest. (# = p < 0.05)

Besides traditional *t*-test for statistical analysis, we added estimation statistics for all MD assays and two group comparing graphs. In short, ‘estimation statistics’ is a simple framework that—while avoiding the pitfalls of significance testing—uses familiar statistical concepts: means, mean differences, and error bars. More importantly, it focuses on the effect size of one’s experiment/intervention, as opposed to significance testing [97]. In comparison to typical NHST plots, estimation graphics have the following five significant advantages such as (1) avoid false dichotomy, (2) display all observed values (3) visualize estimate precision (4) show mean difference distribution. And most importantly (5) by focusing attention on an **effect size**, the difference diagram encourages quantitative reasoning about the system under study [98]. Thus, we conducted a reanalysis of all of our two group data sets using both standard t-tests and estimate statistics. In 2019, the Society for Neuroscience journal eNeuro instituted a policy recommending the use of estimation graphics as the preferred method for data presentation [99].

### Egg and progeny counting

We performed egg laying assay as previously described [44]. In short, wild type females mated with naïve or experienced males were transferred to a fresh new vial and allowed to lay eggs for 24 hr at 25°C. After 24 hr of egg laying, number of eggs were counted under the stereomicroscope. After we count the number of eggs, we kept vials in 25°C incubator and counted the total number of progenies ecolsed from them.

### Fecundity test by introducing the second male

Basically, we followed the protocols previously described by other group [19]. In short, *se*^*1*^ or CS virgin females were introduced to *se*^*1*^ or CS males either as naïve or experienced condition for 24 hours to be confident of all females’ mating with the first males. Then we introduced the second males for 24 hours. After this treatment, we separated females from second males then counted the number of progenies from females. To confirm that the effect from this fecundity test was not originated from the genotype background, we performed the same experiments by reversing the genotypes of the first and second males (*se*^*1*^ then CS vs. CS vs. *se*^*1*^). We calculated the percentage of progeny either from the first male or the second male by counting the eye color of progeny.

### Single-nucleus RNA-sequencing analyses—data and code availability

snRNAseq dataset analyzed in this paper is published in Li et al., doi:10.1126/science.abk2432 [79] and available at the Nextflow pipelines (VSN, https://github.com/vib-singlecell-nf), the availability of raw and processed datasets for users to explore, and the development of a crowd-annotation platform with voting, comments, and references through SCope (https://flycellatlas.org/scope), linked to an online analysis platform in ASAP (https://asap.epfl.ch/fca).

### Gene expression pattern analyses in different tissues

For the gene expression pattern of the 10 genes involved in SMD in each cell type of leg and other tissues, we used the single-cell RNA-seq data from https://flycellatlas.org [79], and the 10x Genomics stringent loom files were downloaded. The cell types are split by FCA.

The digital expression matrices were analyzed with the Seurat 4.1.0 R package [100]. The dot plots of the 10 genes involved in SMD in each cell type of different tissues were then made using the ‘DotPlot’ function with broad annotation (broad cell types) and the annotation (detailed cell types), respectively.

## Supporting information

S1 Fig General characteristics of ‘Shorter-Mating-Duration (SMD)’ behaviour. (A) Mating duration (MD) assays of Oregon-R males. (B) Locomotion of naïve and experienced male flies were quantified as velocity by locomotion activity by horizontal paradigm, and (C) stop frequency by horizontal paradigm. See **EXPERIMENTAL PROCEDURES** section for detailed methods. (D-F) MD assays of CS males after isolated from female experience. Males were reared with sufficient numbers of virgin females for 24 h to be assured having sexual experience then isolated. Assay times after isolation are below the boxes as (D) no recovery, (E) 24 h recovery, and (F) 48 recoveries. (G) The diagram of MD assays of CS males after different time of isolation after sexual experience with females. (H-K) MD assays of CS males after sperm deleted as shown in (L). (M) Histogram of SMD behavior shown in Fig 1B. (N) Normal QQ plot of SMD behavior shown in Fig 1B.
(EPS)

S2 Fig Sensory inputs required for inducing SMD behavior. (A) MD assays of *UAS-KCNJ2* crossed with *Orco-GAL4*. (B) MD assays of *Poxn-RNAi* crossed with *Poxn-GAL4*. (C) MD assay of CS males experienced with *D*. *simulans* females. (D) CS male court oenocytes-musicalized female and show orientation behavior, (E) chasing (F) licking (G) wing extension, (H) copulation attempt, and (I) can successfully mate with it. (J) CS male court feminized male and show wing extension behavior and (K) copulation attempt. (L) MD assays of CS males with oenocytes-masculinized female as a female partner to test whether genotypes of female partners affect MD.
(EPS)

S3 Fig The foreleg of male *Drosophila melanogaster* (A) The anatomical structures of male foreleg are labeled. Ta1-Ta5 comprise fore tarsus and represents tarsomeres 1–5, respectively.
(EPS)

S4 Fig Control experiments for MD assays in **Fig 4** and the location of *Gr5a*-positive neurons in male foreleg (A-D) MD assays of (A) *UAS-Hid/rpr* (B) *UAS-TNT*, *tub-GAL80*^*ts*^ (C)*UAS-KCNJ2*, *tub-GAL80*^*ts*^ (D) *UAS-NachBac*, *tub-GAL80*^*ts*^ crossed with CS. (E) Foreleg tarsus of male flies expressing *Gr5a-lexA* together with *lexAOP-mCherry*. White arrows indicate *Gr5a-*positive neurons and numbers represent the order from the distal part of the leg. (F) Foreleg tarsus (left panels) and tibia (right panels) of male (top panels) or female (bottom panels) flies expressing *Gr5a-GAL4* together with *UAS-RedStigner*. White arrows indicate *Gr5a-*positive neurons. White arrows with dotted line indicate missing neurons in female leg compared to male leg.
(EPS)

S5 Fig (A) Brains of male flies expressing *Gr5a-GAL4* together with *UAS>stop>nSybGFP; fru*^*FLP*^ were immunostained with anti-GFP (green) and nc82 (magenta) antibodies. Scale bars represent 100 μm in the colored panels and 10 μm in the grey panels. White boxes indicate the magnified regions of interest presented next right panels. The right panels are presented as grey scale for clearly showing the axon projection patterns of gustatory neurons in the adult subesophageal ganglion (SOG) labeled by *GAL4* drivers. (B-D) Control experiments for MD assays in **Fig 5.** MD assays of (B) *UAS>stop>TNT*_*active*_*; fru*^*FLP*^ (C) *UAS>stop>TNT*_*inactive*_*; fru*^*FLP*^ (D) *UAS-tra*^*F*^ crossed with CS. (E) Proboscis of male flies expressing *fru-lexA; lexAOP-tdTomato*.*nls*, *UAS-Stinger* with *Gr5a-GAL4* were imaged in live. Scale bars represent 100 μm.
(EPS)

S6 Fig (A) Control experiments for MD assays in **Figs 6B–6D** and **S6B–S6G**. (B-G) MD assays of flies expressing *fru-GAL4* driver together with (B) *Gr61a-RNAi* (C) *Gr64a-RNAi* (D) *Gr64b-RNAi* (E) *Gr64c-RNAi* (F) *Gr64d-RNAi* (G) *Gr64e-RNAi*. (H) Female foreleg (upper panels), midleg (middle panels), and hindleg (bottom panels) of flies expressing *Gr5a-lexA* and *Gr64f-GAL4* drivers together with *lexAOP-tdTomato* and *UAS-Stinger* were imaged live under the fluorescent microscope. Yellow arrows indicate *Gr5a-*positive and *Gr64f*-positive neurons. Scale bars represent 50 mm. (I) Male proboscis of flies expressing *fru-lexA* and *Gr64f- GAL4* drivers together with *lexAOP-tdTomato* and *UAS-Stinger* were imaged live under the fluorescent microscope. Tested gustatory sugar receptors were selected based on previous study [55].
(EPS)

S7 Fig (A) Control experiments for MD assays in **Fig 7A and 7B**. (B-C) Control experiments for MD assays in **Fig 7C–7H**. (D) Female foreleg of flies expressing *Gr5a-lexA* and *Snmp1-GAL4* drivers together with *lexAOP-tdTomato* and *UAS-Stinger* were imaged live under the fluorescent microscope. Scale bars represent 50 μm. (E-F) MD assays for *GAL4* mediated knockdown of LUSH via different *lush-RNAi* using *Gr5a-GAL4*. The stock numbers are written at the bottom of each graph. (G-H) MD assays for *GAL4* mediated knockdown of SNMP1 via different *snmp1-RNAi* using (G) *Gr5a-GAL4* or (H) *Gr66a-GAL4*.
(EPS)

S8 Fig (A-C) Control experiments for MD assays in **Fig 8A–8F**. (D) Male foreleg (upper panels), midleg (middle panels), and hindleg (bottom panels) of flies expressing *Gr5a-lexA* and *ppk23-GAL4* drivers together with *lexAOP-tdTomato* and *UAS-Stinger* were imaged live under the fluorescent microscope. Scale bars represent 50 μm. (H) Control experiments for MD assays in **S8I–S8J Fig**. (I-J) MD assays for *GAL4* mediated knockdown of PPK28 via *ppk28-RNAi* using (I) *Gr5a-GAL4* and (J) *Gr66a-GAL4* drivers.
(EPS)

S9 Fig (A) Male foreleg of flies expressing *ppk29-GAL4* together with *UAS-RedStinger; UAS>stop>mCD8GFP; fru*^*FLP*^ were imaged live under the fluorescent microscope. Scale bars represent 50 μm. (B) Male foreleg of flies expressing *ppk29-GAL4* together with *UAS-RedStinger*, *UAS-mCD8GFP* were imaged live under the fluorescent microscope. Scale bars represent 50 μm. (C) Female foreleg of flies expressing *ppk29-GAL4* together with *UAS-RedStinger*, *UAS-mCD8GFP* were imaged live under the fluorescent microscope. Scale bars represent 50 μm. (D-H) MD assays for *GAL4* mediated knockdown of SNMP2 via *snmp2-RNAi* using (D) *ppk23-GAL4*, (E) *ppk25-GAL4*, (F) *ppk29-GAL4*, (G) *Gr5a-GAL4*, and (H) *Gr64f-GAL4* drivers. (I) MD assays for *GAL4* mediated knockdown of SNMP1 via *snmp1-RNAi* using *ppk25-GAL4*.
(EPS)

S10 Fig (A) A diagram of the cells in the male legs expressing genes involved in SMD behavior.
(EPS)

S11 Fig **(A)** Percentage of progeny originated from *sepia* (*se*) male vs. *CS* male. *CS* male was introduced to *se* female as first mate then followed *se* males as second mate. The eye color of progeny was counted and interpreted as the source of farther; for detailed methods, see **EXPERIMENTAL PROCEDURES**. (B-C) MD assays for *GAL4* mediated knockdown of PPK29 via *ppk29-RNAi* using *elav*^*c155*^ for (B) LMD and (C) SMD behavior.
(EPS)

S12 Fig (A-N) MD assay for *GAL4*, *lexA*, and *RNAi* control experiments. Genotypes are labelled below the graph.
(EPS)

S13 Fig (A-H) MD assay for *GAL4*, *RNAi*, and *UAS-dicer* control experiments. Genotypes are labelled below the graph.
(EPS)

S1 Table Summary of MD assay results with various conditions and sensory mutants to identify the sensory modality for SMD behavior. (DOCX)

S2 Table Summary of MD assay results with various genotypes of experienced females. (DOCX)

S3 Table Summary of normality and lognormality tests of the mating duration result of *Canton S* naïve and exp. condition. (TIF)

S1 Box Costs and benefits of mate guarding. (TIF)

S1 Raw Data All mating duration data generated in this study. (ZIP)

S2 Raw Data All offspring data shown in Fig 11 and S11 Fig. (ZIP)
