# Supplementary material for: Taste and pheromonal inputs govern the regulation of time investment for mating by sexual experience in male Drosophila melanogaster
Source: PLoS Genet. 2023 May 22;19(5):e1010753. doi: 10.1371/journal.pgen.1010753 (PMC10237673; doi:10.1371/journal.pgen.1010753)
Supplement: S1 Table — (DOCX) [file pgen.1010753.s014.docx]

**Table S1.** Summary of MD assay results with various conditions and sensory mutants to identify the sensory modality for SMD behavior

| **Name** | **Genotype of male** | **Description** | **Sensory modalities removed from males** | **Sensory modalities intact in males** | **SMD** | **References** |
| --- | --- | --- | --- | --- | --- | --- |
| *constant*  *dark* | *Canton S* | Wild type males were experienced with wild type females in complete dark condition for overnight. | vision | olfaction/  gustation/  audition/  mechanosensation | normal | [1] |
| *GMR-Hid* | *GMR-Hid* | Expression of apoptotic gene Hid under the GMR promoter which remove the death of the most of photoreceptor expressing compound eye cells. Males are blind. | vision | olfaction/  gustation/  audition/  mechanosensation | normal | [1] |
| *ninaE^17^* | *ninaE^17^* | Mutant male lacks opsin R1-6 photoreceptors | vision | olfaction/  gustation/  audition/  mechanosensation | normal | [1,2] |
| *Orco^1^/*  *Orco^2^* | *Orco^1^/*  *Orco^2^* | Mutant male shows no behavioral or electrophysiological responses to many odorants | olfaction | vision/  gustation/  audition/  mechanosensation | normal | [3] |
| *Olfaction*  *mutant* | *Orco-GAL4/*  *UAS-KCNJ2* | Expressing the KCNJ2 potassium channel in all Orco-expressing olfactory sensing neurons to inactivate Orco-positive neuronal activity | olfaction | vision/  gustation/  audition/  mechanosensation | normal | [1] |
| *gustD^x6^* | *gustD^x6^* | Adults rearing in 28°C shows aberrant gustatory responses. | gustation | vision/  olfaction//  audition/  mechanosensation | impaired | [4] |
| *Poxn mutant* | *Poxn-GAL4/*  *Poxn-RNAi* | Silencing of *Poxn* in all *Poxn*-expressing cells caused all taste bristles in the sensory organ to be transformed into mechanosensory bristles | gustation | vision/  olfaction//  audition/ | impaired | [5] |
| *iav^1^* | *iav^1^* | Abnormal auditory perception, mechanosensory responses and thermosensory responses | audition/  mechanosensation/  thermosensation | vision/  olfaction//  gustation | impaired | [6–9] |

**Reference**

1. Kim WJ, Jan LY, Jan YN. Contribution of visual and circadian neural circuits to memory for prolonged mating induced by rivals. Nat Neurosci. 2012;15: 876–883. doi:10.1038/nn.3104

2. Cook T, Pichaud F, Sonneville R, Papatsenko D, Desplan C. Distinction between Color Photoreceptor Cell Fates Is Controlled by Prospero in Drosophila. Dev Cell. 2003;4: 853–864. doi:10.1016/s1534-5807(03)00156-4

3. Benton R, Sachse S, Michnick SW, Vosshall LB. Atypical Membrane Topology and Heteromeric Function of Drosophila Odorant Receptors In Vivo. Plos Biol. 2006;4: e20. doi:10.1371/journal.pbio.0040020

4. Rodrigues V, Sathe S, Pinto L, Balakrishnan R, Siddiqi O. Closely linked lesions in a region of the X chromosome affect central and peripheral steps in gustatory processing in Drosophila. Mol Gen Genetics Mgg. 1991;226–226: 265–276. doi:10.1007/bf00273612

5. Raad H, Ferveur J-F, Ledger N, Capovilla M, Robichon A. Functional Gustatory Role of Chemoreceptors in Drosophila Wings. Cell Reports. 2016;15: 1442–1454. doi:10.1016/j.celrep.2016.04.040

6. Bretman A, Westmancoat JD, Gage MJG, Chapman T. Males Use Multiple, Redundant Cues to Detect Mating Rivals. Curr Biol. 2011;21: 617–622. doi:10.1016/j.cub.2011.03.008

7. Gong Z, Son W, Chung YD, Kim J, Shin DW, McClung CA, et al. Two Interdependent TRPV Channel Subunits, Inactive and Nanchung, Mediate Hearing in Drosophila. J Neurosci. 2004;24: 9059–9066. doi:10.1523/jneurosci.1645-04.2004

8. Kwon Y, Shen WL, Shim H-S, Montell C. Fine Thermotactic Discrimination between the Optimal and Slightly Cooler Temperatures via a TRPV Channel in Chordotonal Neurons. J Neurosci. 2010;30: 10465–10471. doi:10.1523/jneurosci.1631-10.2010

9. Agrawal S, Dickinson ES, Sustar A, Gurung P, Shepherd D, Truman JW, et al. Central processing of leg proprioception in Drosophila. Elife. 2020;9: e60299. doi:10.7554/elife.60299
