# Supplementary material for: Taste and pheromonal inputs govern the regulation of time investment for mating by sexual experience in male Drosophila melanogaster
Source: PLoS Genet. 2023 May 22;19(5):e1010753. doi: 10.1371/journal.pgen.1010753 (PMC10237673; doi:10.1371/journal.pgen.1010753)
Supplement: S2 Table — (DOCX) [file pgen.1010753.s015.docx]

**Table S2.** Summary of MD assay results with various genotypes of experienced females

| **Name** | **Genotype of**  **female** | | **Description** | **Experiences that were removed or provided from this genotype of female** | **Sensory cues still can be provided from female** | **SMD** | **References** |
| --- | --- | --- | --- | --- | --- | --- | --- |
| *mated* | *Canton S* | | Canton S virgin females were mated with twice number of males for more than 4 hours to be surely mated. Mated females reject the male’s copulation attempt in most of cases. | Remove  copulation experiences | Female looks/  Female sound/  Female pheromone | impaired | [1] |
| *D. pse*  *female* | *Drosophila*  *pseudobscura* | | *D. melanogaster* males cannot mate with *D. pseudobscura* females | Remove  all the cues from  *D. melanogaster* females | N/A | impaired | [2] |
| *D. sim*  *female* | *Drosophila*  *simulans* | | *D. melanogaster* males can mate with *D. simulans* females and produce hybrid offsprings | Provide similar sensory cues of D. melanogaster females | Female looks/  Female sound/  Female pheromone | normal | [3] |
| *Def^exel6234^* | *Def^exel6234^* | | Genomic region contains SPR deleted, which let this female mate multiple times with males | Provide  multiple mating experiences* | Female looks/  Female sound/  Female pheromone | normal | [1] |
| *fru-GAL4/*  *UAS-mSP* | *fru-GAL4/*  *UAS-mSP* | | Express membrane boud form of sex peptide, which induce post mating responses of females and change virgin female behavior into mated female | Remove  copulation experiences | Female looks/  Female sound/  Female pheromone | impaired | [4,5] |
| *oenocyte (-)* | *oenocyte (-)* | | Oenocyte of females were removed, which eliminate the most of female pheromones produced from oenocyte | Remove  female pheromone | Female looks/  Female sound/ | impaired | [6] |
| *oeno-GAL4/*  *tra-RNAi* | *oeno-GAL4/*  *tra-RNAi* | Masculinize female oenocyte into male form by knockdown *transformer* gene using oenocyte-specific *GAL4* driver | | Remove  female pheromone | Female looks/  Female sound/ | impaired | [4] |
| *elav^c155^/*  *UAS-dicerl;*  *tra-RNAi* | *elav^c155^/*  *UAS-dicerl;*  *tra-RNAi* | Masculinize female neurons into male form by knockdown *transformer* gene using pan-neuronal *elav-GAL4* driver | | Remove  female specific behaviors | Female looks/  Female pheromone | normal | [5] |
| *(male)*  *actin-GAL4/*  *UAS-tra^F^* | *(male)*  *actin-GAL4/*  *UAS-tra^F^* | Feminize all cells of males into female form, which induce males behave and look as female | | Provide  partial copulation experiences | Female looks/  Female sound/  Female pheromone | normal | [4,5] |

* multiple mating of *Df^exel6234^* females with males were confirmed by others [1]

**References**

1. Yang C, Rumpf S, Xiang Y, Gordon MD, Song W, Jan LY, et al. Control of the Postmating Behavioral Switch in Drosophila Females by Internal Sensory Neurons. Neuron. 2009;61: 519–526. doi:10.1016/j.neuron.2008.12.021

2. Fan P, Manoli DS, Ahmed OM, Chen Y, Agarwal N, Kwong S, et al. Genetic and Neural Mechanisms that Inhibit Drosophila from Mating with Other Species. Cell. 2013;154: 89–102. doi:10.1016/j.cell.2013.06.008

3. Wood D, Ringo JM. Male Mating Discrimination in Drosophila melanogaster, D. simulans and Their Hybrids. Evolution. 1980;34: 320. doi:10.2307/2407395

4. Rideout EJ, Dornan AJ, Neville MC, Eadie S, Goodwin SF. Control of sexual differentiation and behavior by the doublesex gene in Drosophila melanogaster. Nat Neurosci. 2010;13: 458–466. doi:10.1038/nn.2515

5. Yamamoto D, Fujitani K, Usui K, Ito H, Nakano Y. From behavior to development: genes for sexual behavior define the neuronal sexual switch in Drosophila. Mech Develop. 1998;73: 135–146. doi:10.1016/s0925-4773(98)00042-2

6. Billeter J-C, Atallah J, Krupp JJ, Millar JG, Levine JD. Specialized cells tag sexual and species identity in Drosophila melanogaster. Nature. 2009;461: 987–991. doi:10.1038/nature08495
