# Supplementary figures and images for: Taste and pheromonal inputs govern the regulation of time investment for mating by sexual experience in male Drosophila melanogaster
Source: PLoS Genet. 2023 May 22;19(5):e1010753. doi: 10.1371/journal.pgen.1010753 (PMC10237673; doi:10.1371/journal.pgen.1010753)

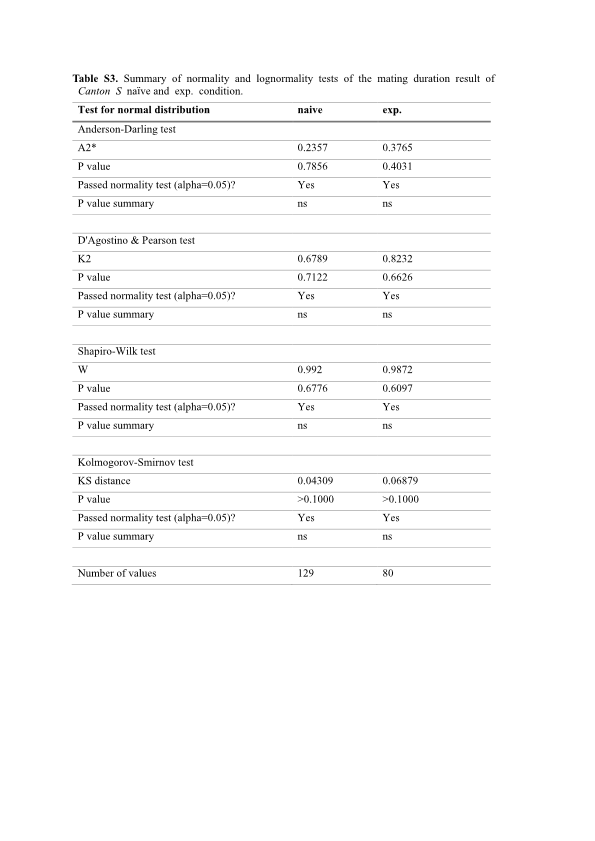

Supplement: S3 Table — (TIF) [file pgen.1010753.s016.tif]

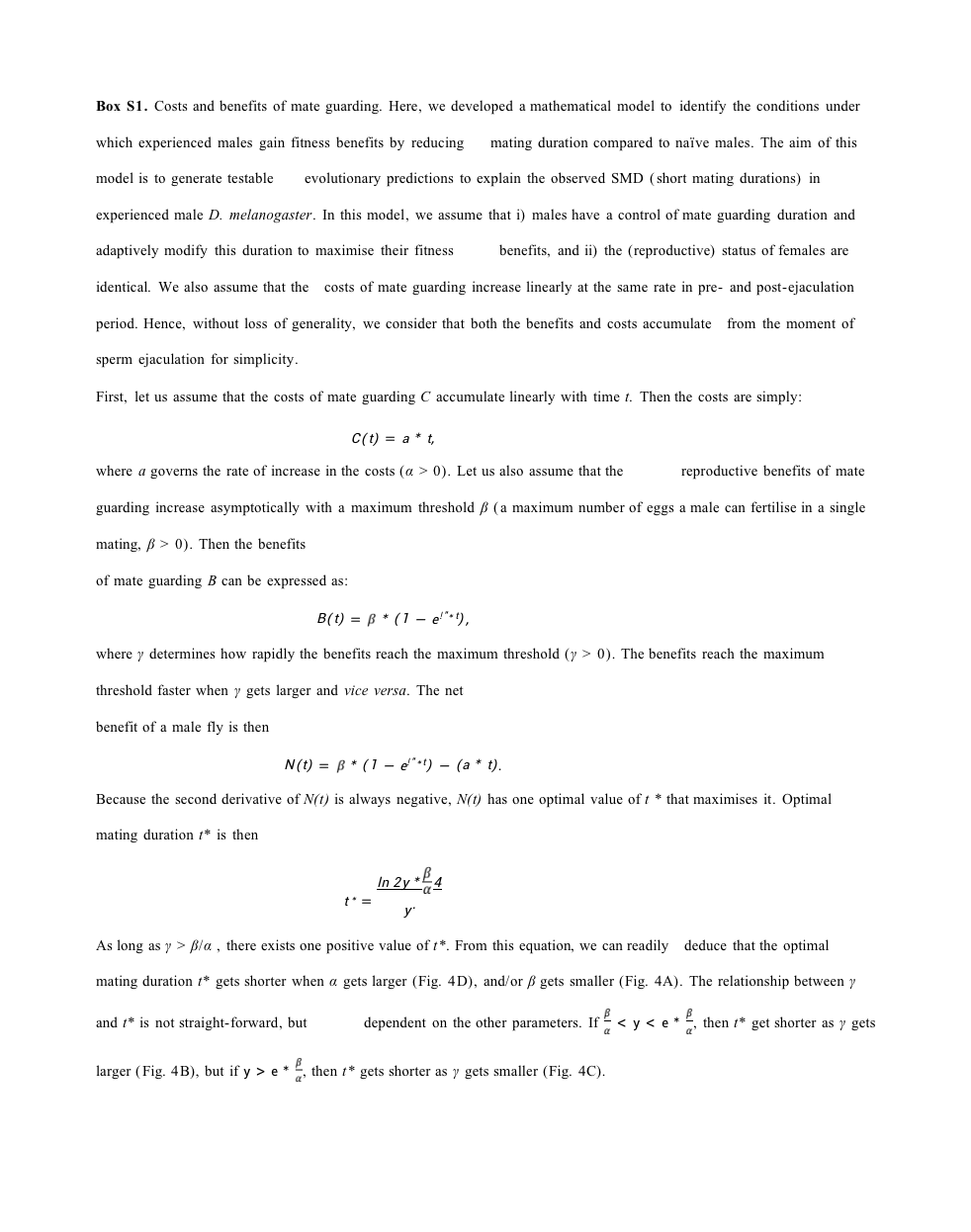

Supplement: S1 Box — (TIF) [file pgen.1010753.s017.tif]
